# Immunochemical characterization on pathological oligomers of mutant Cu/Zn-superoxide dismutase in amyotrophic lateral sclerosis

**DOI:** 10.1186/s13024-016-0145-9

**Published:** 2017-01-05

**Authors:** Eiichi Tokuda, Itsuki Anzai, Takao Nomura, Keisuke Toichi, Masahiko Watanabe, Shinji Ohara, Seiji Watanabe, Koji Yamanaka, Yuta Morisaki, Hidemi Misawa, Yoshiaki Furukawa

**Affiliations:** 1 Laboratory for Mechanistic Chemistry of Biomolecules, Department of Chemistry, Keio University, 3-14-1 Hiyoshi, Kohoku, Yokohama, Kanagawa 223-8522 Japan; 2 Department of Anatomy, Hokkaido University Graduate School of Medicine, Sapporo, 060-8638 Japan; 3 Department of Neurology, Matsumoto Medical Center, Matsumoto, 399-0021 Japan; 4 Department of Neuroscience and Pathobiology, Research Institute of Environmental Medicine, Nagoya University, Nagoya, 464-8601 Japan; 5 Division of Pharmacology, Faculty of Pharmacy, Keio University, Tokyo, 105-8512 Japan

**Keywords:** Amyotrophic lateral sclerosis, Cu/Zn-superoxide dismutase, Protein misfolding, Disulfide bond

## Abstract

**Background:**

Dominant mutations in Cu/Zn-superoxide dismutase (*SOD1*) gene cause a familial form of amyotrophic lateral sclerosis (*SOD1*-ALS) with accumulation of misfolded SOD1 proteins as intracellular inclusions in spinal motor neurons. Oligomerization of SOD1 *via* abnormal disulfide crosslinks has been proposed as one of the misfolding pathways occurring in mutant SOD1; however, the pathological relevance of such oligomerization in the *SOD1*-ALS cases still remains obscure.

**Methods:**

We prepared antibodies exclusively recognizing the SOD1 oligomers cross-linked *via* disulfide bonds in vitro. By using those antibodies, immunohistochemical examination and ELISA were mainly performed on the tissue samples of transgenic mice expressing mutant SOD1 proteins and also of human *SOD1*-ALS cases.

**Results:**

We showed the recognition specificity of our antibodies exclusively toward the disulfide-crosslinked SOD1 oligomers by ELISA using various forms of purified SOD1 proteins in conformationally distinct states in vitro. Furthermore, the epitope of those antibodies was buried and inaccessible in the natively folded structure of SOD1. The antibodies were then found to specifically detect the pathological SOD1 species in the spinal motor neurons of the *SOD1*-ALS patients as well as the transgenic model mice.

**Conclusions:**

Our findings here suggest that the SOD1 oligomerization through the disulfide-crosslinking associates with exposure of the SOD1 structural interior and is a pathological process occurring in the *SOD1*-ALS cases.

**Electronic supplementary material:**

The online version of this article (doi:10.1186/s13024-016-0145-9) contains supplementary material, which is available to authorized users.

## Background

Amyotrophic lateral sclerosis (ALS) is a neurodegenerative disease, which associates with loss of motor neurons in the affected nervous tissues including motor cortex, brainstem, and spinal cords [1]. After several years of disease onset, significant weakness of muscles is usually followed by death due to the failure in respiratory system. While most of the ALS cases are sporadic, dominant mutations in Cu/Zn-superoxide dismutase (*SOD1*) gene have been shown to cause familial forms of ALS (*SOD1*-ALS) [2]. More than 150 types of pathogenic mutations in *SOD1* gene have been identified [3], but importantly, no ALS-like phenotypes were confirmed in *SOD1*-knockout mice [4]. SOD1 is hence considered to gain toxic properties by pathogenic mutations. A common pathological hallmark in *SOD1*-ALS cases is the abnormal accumulation of mutant SOD1 proteins in motor neurons of affected nervous tissues [5]. Pathogenic mutations have hence been proposed to facilitate “misfolding” of SOD1 into abnormal conformation(s) and thereby exert toxicities causing the disease.

SOD1 is a homodimeric metalloprotein that binds copper and zinc ions and also forms an intramolecular disulfide bond [6]. A folded conformation of enzymatically active SOD1 is significantly stabilized through the metal binding and the disulfide formation [7]. Indeed, dissociation of metal ions and reduction of the disulfide bond are known to decrease the conformational stability of SOD1 and thereby facilitate its misfolding in vitro; for example, demetallated (apo) SOD1 forms cross-linked oligomers through the shuffling of the disulfide bond [8], and further reduction of the disulfide bond in apo-SOD1 leads to the formation of the amyloid-like fibrillar aggregates [9]. Abnormal SOD1 trimers have also been recently shown to form in vitro at acidic pH and exhibit toxicities toward cultured cells [10]. Moreover, the structural dynamics of immature SOD1 has been extensively characterized in the atomic level [11–13]. Increasing numbers of recent in vitro studies have revealed various misfolding pathways of SOD1 proteins; however, it still remains obscure how SOD1 changes its conformation under the pathological conditions in vivo.

Actually, quite limited information is available on the biochemical/structural properties of pathological SOD1 species in human *SOD1*-ALS cases, partly because most of the motor neurons, which are the most affected cell types in ALS, are usually lost at autopsies. Therefore, any changes of SOD1 occurring specifically in affected motor neurons could not become evident in the biochemical experiments using the homogenates of spinal cords. Nonetheless, in transgenic ALS-model mice overexpressing mutant SOD1, the enzymatic activation of SOD1 has been shown to be retarded in spinal cords but not in the control tissues such as kidney and liver [14]. In other words, immature forms of SOD1 are expected to accumulate specifically in the spinal cord as misfolded proteins. Actually, the amyloid-like aggregates, which are composed of SOD1 lacking the disulfide bond, have been detected in the spinal motor neurons of transgenic ALS-model mice [15], while there has been no evidence to support the formation of amyloid-like aggregates in human *SOD1*-ALS cases [16]. Alternatively, we have previously detected the disulfide-crosslinked SOD1 oligomers in the spinal cord but not in the liver of ALS-model mice [17]. Pathological roles of the disulfide-crosslinked oligomers have been examined mainly in cultured cells [18, 19] but still remain less characterized in the transgenic mice and also in human *SOD1*-ALS cases.

In this study, we prepared and characterized the antibodies recognizing the disulfide-crosslinked SOD1 oligomers in vitro to test their pathological relevance in *SOD1*-ALS. Compared to the previous studies, we have more extensively examined the reactivity of our antibodies against purified SOD1 proteins in various metallation/disulfide states and ensured the recognition specificities of our antibodies to the disulfide-crosslinked SOD1 oligomers. In the ALS-model mice, the immunoreactivities with our antibodies were evident from their pre-symptomatic stage and also specifically in their spinal cords. More importantly, the immunoreactivities with our antibodies were detected in spinal motor neurons of the human *SOD1*-ALS cases. We thus propose that the disulfide-crosslinked SOD1 oligomers possess an immunological epitope selective for the SOD1 species with abnormal conformations occurring in the pathological conditions.

## Methods

### Protein preparation and purification

Introduction of mutations was performed by an Inverse PCR method using a KOD-FX-neo DNA polymerase (TOYOBO) and confirmed by DNA sequencing. *Escherichia coli* SHuffle™ (NEB) was transformed with a pET-15b plasmid (Novagen) containing cDNA of human SOD1, and the protein expression was induced in the shaking culture with 0.1 mM isopropyl β-D-1-thiogalactopyranoside (IPTG) at 20 °C for 20 h. Cells were lysed with ultrasonication in PBS containing 2% Triton X-100, DNase I, and MgSO_4_, and the supernatant after centrifugation at 20,000 x *g* for 15 min. was loaded on a HisTrap HP column (1 mL, GE Healthcare). SOD1 proteins were eluted with a buffer containing 50 mM sodium phosphate (Na-Pi), 100 mM NaCl, and 250 mM imidazole at pH 7.0. Metal ions bound to SOD1 proteins were removed by two-step dialysis first against a buffer containing 50 mM sodium acetate, 100 mM NaCl, and 10 mM EDTA at pH 4.0 at 4 °C for 16 h and then against a buffer containing 100 mM Na-Pi, 100 mM NaCl, and 5 mM EDTA at pH 7.4 (called NNE buffer). The proteins were treated with thrombin (GE Healthcare) to remove an N-terminal His-tag and further purified by size-exclusion chromatography using a Cosmosil 5Diol-300-II column (nacalai tesque).

For the epitope mapping of antibodies, we have prepared the following eight peptides as a fusion protein with glutathione-S-transferase, which was further N-terminally tagged with a 6 x His tag: Ala 1 – Lys 23 (Pep^exon1^), Glu 24 – Ala 55 (Pep^exon2^), Gly 56 – Arg 79 (Pep^exon3^), His 80 – Val 118 (Pep^exon4^), Val 119 – Gln 153 (Pep^exon5^), Glu 24 – His 43 (Pep1), Ser 34 – Asn 53 (Pep2), and Gly 44 – His 63 (Pep3). All of those fusion proteins were overexpressed in *E. coli* BL21(DE3) with shaking at 20 °C for 20 h in the presence of 0.1 mM IPTG. As described above, the cells were lysed, and the fusion proteins were purified from the soluble supernatant with a HisTrap HP column.

### Preparation and purification of anti-SOD1^olig^ antibody

Demetallated SOD1 with A4V mutation as purified above (5 mg/mL) was incubated in the NNE buffer at 37 °C for five days, by which soluble SOD1(A4V) oligomers were prepared. Those SOD1(A4V) oligomers were emulsified with either complete Freund's adjuvant (DIFCO) in the initial injection or incomplete Freund's adjuvant and injected subcutaneously into a female New Zealand White rabbit at intervals of 2 – 4 weeks. Antisera were sampled at two weeks after the fifth or sixth injection, and immunoglobulins specific to antigen were affinity-purified using CNBr-activated Sepharose 4B (GE Healthcare) conjugated with the SOD1(A4V) oligomers.

To isolate the antibodies recognizing SOD1 oligomers but not the folded proteins, the affinity-purified immunoglobulins were washed with Ni^2+^-affinity resins that bind His-tagged wild-type SOD1^S-S^ proteins (SOD1-resins). SOD1-resins were prepared by adding 100 μL of His-SELECT nickel affinity gel (Sigma) to 500 μL of 200 μM His-tagged wild-type SOD1^S-S^ in a buffer containing 50 mM Tris and 100 mM NaCl at pH 7.4 and incubated at 4 °C for an hour. The resins were washed with PBS and then incubated with the affinity-purified immunoglobulins in PBS with rotation at 4 °C for an hour. The resins were spun down, and again, the freshly prepared SOD1-resins were added to the supernatant and rotated at 4 °C for an hour. After repeating this absorption procedure four times, Ni^2+^-affinity resins were added to the supernatant in order to remove His-tagged SOD1 proteins detached from the SOD1-resins. Concentrations of purified antibodies were then determined by Micro BCA Protein Assay kit (Thermo).

### Preparation and purification of anti-SOD1^int^ antibody

Production of a polyclonal antibody to a peptide of SOD1 (Gly 44 – Asn 53) was performed by Eurofins Genomics. Briefly, the peptide, H_2_N-CG^44^FHVHEFGDN^53^-COOH, was conjugated through its N-terminal Cys with keyhole limpet hemocyanin, with which a rabbit was immunized in the 42-day protocol. The sera were then purified using a Sulfo-Link Coupling Resin (Thermo) with the peptide, Gly 44 – Asn 53, Gly 44 – Glu 49, His 46 – Gly 51, or His 48 – Asn 53, by which anti-SOD1^44–53^, anti-SOD1^44–49^, anti-SOD1^46–51^, or anti-SOD1^48–53^ antibody was purified, respectively. All of the peptides have an additional Cys residue at the N-terminus for its conjugation with the resin. For preparation of anti-SOD1^int^ antibody, anti-SOD1^44–53^ antibody was first loaded on a Sulfo-Link Coupling Resin (Thermo) cross-linked with purified apo-SOD1^S-S^ proteins, and the flow-through fraction was collected, concentrated, and then purified using a Sulfo-Link resin conjugated with a His 48 – Asn 53 peptide. Concentrations of purified antibodies were determined by Micro BCA Protein Assay kit (Thermo).

### Enzyme-linked immunosorbent assay (ELISA)

To prevent adventitious binding of contaminant metal ions to SOD1 proteins in ELISA, we used Tris-buffered saline (TBS) that was treated with Chelex® 100 Resin (Bio-Rad). For the assay of E,E-SOD1 proteins, a strong chelator for divalent metal ions, EDTA (5 mM), was further included in TBS, by which an artificial supply of divalent metal ions (zinc ions, in particular) from buffers could be prevented. SOD1 variants with distinct metallation and thiol-disulfide status (5 μg/well) were coated on 96-well plates (Nunc-Immuno™ Plate CII, Thermo) overnight at 4 °C. After three washes with TBS containing 0.05% (v/v) Tween 20 (TBS-T), the plates were blocked with TBS containing 0.5% (w/v) BSA for an hour at room temperature. After six washes with TBS-T, either antibody purified in this study, polyclonal anti-human SOD1 (FL-154, Santa Cruz Biotechnology), USOD (#SPC-205, StressMarq Bioscience), or SEDI (#SPC-206, StressMarq Bioscience) antibody was added as a primary antibody (0.2 μg/mL) and incubated for an hour at room temperature, which was then followed by secondary antibody with horseradish peroxidase (goat anti-rabbit IgG, 1:1,000; Thermo Scientific) for an hour at room temperature. As the substrate solution, *O*-phenylenediamine and 0.012% H_2_O_2_ in a buffer containing 100 mM sodium citrate at pH 5.0 were used. The absorbance was read at 490 nm using a plate reader (Epoch, BioTek).

For sandwich ELISA, a plate (Nunc-Immuno™ Plate CII, Thermo) was coated with the capture antibodies (0.2 μg/mL anti-SOD1^int^ or 0.02 μg/mL anti-SOD1 (FL-154, Santa Cruz Biotechnology) antibodies) overnight at 4 °C and blocked with 1% BSA for an hour. Soluble extracts of the tissue samples containing 10 μg of total proteins were then applied and incubated at room temperature for an hour. The captured SOD1 proteins were detected by sheep anti-SOD1 (1:2,000, Calbiochem) and HRP-conjugated rabbit anti-sheep (1:1,000, Bio-Rad) antibodies as the detection and secondary antibodies, respectively. As the substrate solution, *O*-phenylenediamine and 0.012% H_2_O_2_ in a buffer containing 100 mM sodium citrate at pH 5.0 were used. The absorbance was read at 490 nm using a plate reader (Epoch, BioTek).

### Transgenic mice

Transgenic mice carrying human *SOD1* gene with G93A mutation (B6.Cg-Tg(SOD1*G93A)1Gur/J in a C57BL/6 background) and human wild-type *SOD1* gene (B6.Cg-Tg(SOD1)2Gur) were purchased from Jackson Laboratory (Bar Harbor, ME) and maintained heterozygous with a C57BL/6 background. Mice expressing human SOD1 with G37R were described previously [20]. Mice were genotyped for human *SOD1* using tail DNA as described previously [21]. All experiments were reviewed and approved by the Animal Use and Care Committees of Keio University and Nagoya University, and care was taken to minimize suffering and limit the number of animals used.

Mice were deeply anesthetized with sodium pentobarbital and then perfused *via* the aortic cone with PBS, followed by 4% paraformaldehyde in a buffer containing 0.1 M Na-Pi at pH 7.4. The lumbar region of each spinal cord (*ca*. 2 cm) was removed and post-fixed in the same fixative overnight at 4 °C, after which it was immersed in 20% sucrose in 0.1 M Na-Pi, pH 7.4, overnight at 4 °C. The tissue was then frozen in OCT compound (Sakura Finetek) and sectioned at 40 μm on a cryostat. Rabbit anti-SOD1^olig^ (0.02 μg/mL) and mouse monoclonal anti-human SOD1 (0.02 μg/mL, clone 1G2, MBL) antibodies were used for immunohistochemistry as a primary antibody, and biotinylated anti-mouse IgG (H + L) (1:200 dilution, Vector Laboratories, Inc.) was used as a secondary antibody. The immunoreaction was amplified using the VECTASTAIN ABC HRP Kit (Vector Laboratories, Inc.) according to the manufacturer’s direction. The free-floating sections were processed using diaminobenzidine (DAB) as the chromogen followed by counter-staining with hematoxylin [22]. Stained sections were then examined using a microscope (BX51, Olympus).

### Human cases

The human cases examined in this study included three *SOD1*-ALS cases with C111Y mutation, four sporadic ALS cases with TDP-43-positive inclusions (negative for SOD1 mutations), and three non-ALS controls. All tissues from ALS patients and non-ALS controls were obtained by autopsy with informed consent at Matsumoto Medical Center in Japan, and information on the cases was summarized in Additional file 1: Table S1. The collection of tissues and their use in this study were approved by the institutional review board for research ethics of Matsumoto Medical Center and Keio University, Japan.

For immunohistochemical examination, the spinal cord was fixed in 10% buffered formalin, and multiple tissue blocks were embedded in paraffin. Deparaffinized 4-*μ*m-thick sections were immunostained by the streptavidin-biotin method using rabbit anti-SOD1^olig^ (0.02 μg/mL), rabbit anti-SOD1^int^ (0.3 μg/mL), mouse monoclonal anti-human SOD1 (0.5 μg/mL, clone 1G2, MBL) antibodies, and the corresponding biotin-conjugated secondary antibodies. The sections were processed with HRP-conjugated streptavidin and DAB as the chromogen and further stained for nuclei with hematoxylin. For double immunofluorescence, deparaffinized sections were first incubated with Sudan Black B to suppress auto-fluorescence and then stained with the primary antibodies followed by the corresponding FITC- or Cy3-labeled secondary antibodies (Jackson Labs, Pittsburgh, PA).

### Sample preparations for biochemical analysis on human and mouse tissues

For human cases, the ventral and dorsal horns were separately excised from the frozen thoractic spinal cord samples. Frozen mice tissues (lumbar spinal cord, cervical spinal cord, cerebellum, and brainstem) were also separately prepared. The tissues were then homogenized and ultrasonicated in PBS containing 1% NP-40, 100 mM iodoacetamide, 5 mM EDTA, and EDTA-free Complete Protease inhibitor cocktail (Roche). The homogenates were centrifuged (20,000 x *g*, 30 min. 4 °C) to prepare the soluble supernatants and then examined for their total protein concentrations by using Micro BCA Assay Kit (Thermo Scientific).

### Western blotting analysis

Soluble proteins in the tissue extracts (15 μg/lane) were separated using 12.5% polyacrylamide gels and blotted onto a PVDF membrane (0.2 μm, Wako). The membrane was treated with a blocking solution containing 5% (w/v) dried milk and 0.01% (v/v) Tween 20 in PBS at pH 7.4. The blots were probed with rabbit anti-SOD1 antibody (1:10,000; FL-154, Santa Cruz Biotechnology) and HRP-conjugated goat anti-rabbit IgG antibody (1:10,000; Thermo Scientific), visualized using ImmunoStar LD (Wako), and then observed in the LumiCube (Liponics). To validate an equal loading of tissue extracts, glyceraldehyde 3-phosphate dehydrogenase (GAPDH) was used as an internal marker. The membranes were treated with the WB Stripping Solution (nacalai tesque) for 1 h at 37 °C and reprobed with rabbit anti-GAPDH antibody (1:5,000; FL-335, Santa Cruz Biotechnology).

### Statistics

All statistical tests were performed using Statcel 3 software (OMS Publishing Inc.). After the determination of normality, multiple group comparisons were performed using a one-way ANOVA followed by the Tukey–Kramer *post-hoc* test.

## Results

### Preparation of soluble and disulfide-crosslinked oligomers of SOD1 in vitro

**Fig. 1:**
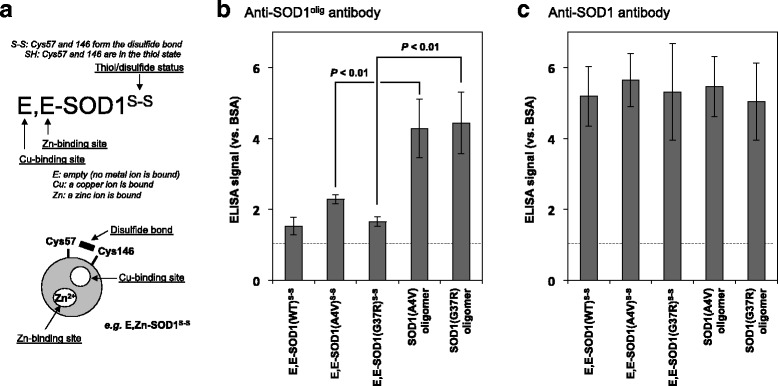
Anti-SOD1^olig^ antibody recognizes soluble SOD1 oligomers with the disulfide crosslinks but not folded SOD1 in vitro. (**a**) Description of SOD1 in distinct metallation and thiol-disulfide states. E,Zn-SOD1^S-S^, in which SOD1 with the disulfide bond binds a zinc ion at the Zn-binding site, is shown as an example. (**b**) Specificity of purified anti-SOD1^olig^ antibody was examined by indirect ELISA using E,E-SOD1^S-S^ proteins (WT, A4V, G37R) and soluble disulfide-crosslinked oligomers (A4V, G37R). (**c**) No statistically significant difference in the amounts of SOD1 proteins adsorbed on wells was confirmed by ELISA using anti-SOD1 antibody (FL-154, Santa Cruz Biotechnology). The ELISA signal was represented as a ratio against that obtained using bovine serum albumin (BSA). Three independent experiments were performed to estimate error bars (standard deviation)

Among four Cys residues (Cys 6, 57, 111, and 146) in SOD1, the conserved disulfide bond forms between Cys 57 and 146 within an SOD1 molecule and significantly contributes to structural stabilization of a SOD1 protein [7]. Under destabilizing conditions in vitro, however, the disulfide bond of SOD1 is shuffled among the four Cys residues in inter- as well as intra-molecular fashion, resulting in the formation of SOD1 oligomers cross-linked *via* disulfide bond(s) [8]. In this study, an apo and disulfide form of SOD1 (E,E-SOD1^S-S^; the first and second E mean empty at copper and zinc sites, respectively; thiol-disulfide status is indicated as superscript.) (Fig. 1a) was first prepared and then incubated at 37 °C for five days without any agitation. SOD1 with ALS-causing mutations formed the high-molecular weight species, which remained in supernatant after centrifugation at 20,000 x *g* and were observed in non-reducing but not in reducing SDS-PAGE (Additional file 2: Figure S1). These results confirm the formation of the soluble SOD1 oligomers cross-linked *via* disulfide bonds.

### Purification of an antibody recognizing the disulfide-crosslinked SOD1 oligomers

A rabbit was first immunized with the soluble and disulfide-crosslinked oligomers of A4V-mutant SOD1 prepared in vitro, and then the polyclonal antibodies were affinity-purified using those oligomers. Nonetheless, the purified antibody was not selective to the oligomers; a natively folded SOD1 protein (Cu,Zn-SOD1^S-S^) significantly reacted with the antibody (data not shown). To increase the specificity of antibodies to the oligomers, the purified antibodies were washed with Ni^2+^-affinity resins on which wild-type SOD1^S-S^ was immobilized through its N-terminal His tag (see Methods). As shown in Fig. 1b and c, the finally purified antibody (called anti-SOD1^olig^ antibody) exhibited significantly higher ELISA signals to the soluble disulfide-crosslinked SOD1(A4V) oligomer than those to wild-type (WT) and A4V-mutant E,E-SOD1^S-S^ proteins (*P* < 0.01). It is also important to note that anti-SOD1^olig^ antibody can recognize the soluble and disulfide-crosslinked oligomers of G37R-mutant SOD1 but not E,E-SOD1(G37R)^S-S^ (*P* < 0.01: Fig. 1b and c). Our anti-SOD1^olig^ antibody was hence found to exclusively recognize the soluble and disulfide-crosslinked SOD1 oligomers.

### Immunohistochemical detection of pathological SOD1 species by anti-SOD1^olig^ antibody

**Fig. 2:**
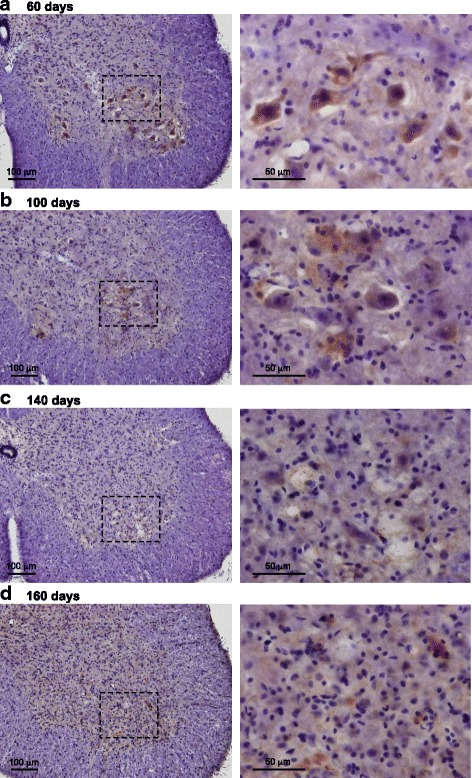
Immunohistochemical examination on G1H mice with anti-SOD1^olig^ antibody. The sections of lumbar spinal cords (ventral horn) of G1H mice at **a** 60, **b** 100, **c** 140, and **d** 160 days of age were stained with anti-SOD1^olig^ antibody. The images in the low magnification are shown in the left panel, where the region enclosed with a broken line is magnified and shown in the right panel. Nuclei were counterstained with hematoxylin (blue). The bar in each panel represents 100 μm (left panel) and 50 μm (right panel)

We previously detected the disulfide-crosslinked SOD1 species by isolating those from the spinal cord homogenates of ALS-model mice [17, 23]. In this study, we attempted to probe the disulfide-crosslinked oligomers during the pathogenesis of *SOD1*-ALS by using our anti-SOD1^olig^ antibody. For that purpose, the ALS-model mice expressing human SOD1 with G93A mutation (G1H mice) on a congenic C57BL/6 background were immunohistochemically examined. As shown in Fig. 2a and b, the species immunoreactive to anti-SOD1^olig^ antibody were observed in the ventral horn of the lumbar spinal cord before the disease onset (at 60 and 100 days of age). No staining with anti-SOD1^olig^ antibody was confirmed in the corresponding area of spinal cords of a non-transgenic mouse (Additional file 3: Figure S2A and B). Also, when anti-SOD1^olig^ antibody was pre-absorbed with the disulfide-crosslinked oligomers of A4V-mutant SOD1, any immunoreactive species were not observed in the spinal cord of a G1H mouse (100 days) (Additional file 3: Figure S2C). Instead, when pre-adsorbed with E,Zn-SOD1(A4V)^S-S^, anti-SOD1^olig^ antibody was able to detect the immunoreactive species in the spinal cord of a G1H mouse (100 days) (Additional file 3: Figure S2D). These control experiments assure the specificity of anti-SOD1^olig^ antibody toward the pathological SOD1 species in the transgenic mice.

At the end stage of the disease (140 and 160 days of age), however, immunostaining with anti-SOD1^olig^ antibody was significantly reduced in the lumbar spinal cord of G1H mice (Fig. 2c and d),. This was not described by the changes in total amounts of soluble SOD1 in the lumbar spinal cord, which was actually increased during aging ([14]; also see below). We previously showed that the number of the ChAT-positive motor neurons at 140 days of age was reduced down to one third of those at 60 days of age in G1H mice [24]. The reduced immunostaining with anti-SOD1^olig^ antibody might thus indicate loss of motor neurons at the disease end-stage. Nonetheless, mutant SOD1 has been also known to accumulate as inclusions in the surviving motor neurons of diseased G1H mice [25]. Actually, the diffuse staining of SOD1 was observed in the lumbar spinal cord of pre-symptomatic G1H mice (60 and 100 days of age), and the SOD1-positive inclusions became evident in the disease end-stage (140 and 160 days of age) (Additional file 4: Figure S3). Our anti-SOD1^olig^ antibody is hence expected to have little immunoreactivity toward the SOD1-positive inclusions formed in terminally ill G1H mice. Based upon these results, we suggest that anti-SOD1^olig^ antibody specifically detects the pathological SOD1 species occurring in the lumbar spinal cords of the pre-symptomatic G1H mice.

**Fig. 3:**
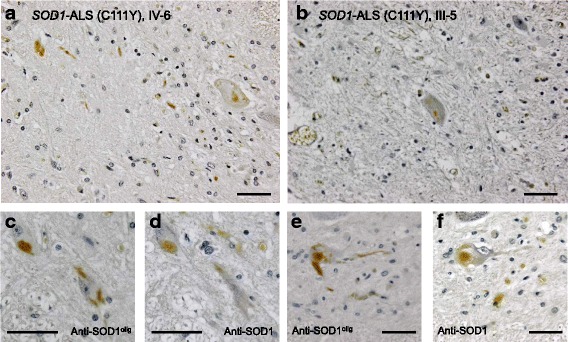
Immunohistochemical examination of human *SOD1*-ALS cases (C111Y mutation) with anti-SOD1^olig^ antibody. DAB staining of **a** a sacral spinal cord (ventral horn) section of the case IV-6 and **b** a cervical spinal cord (ventral horn) of the case III-5 was performed using anti-SOD1^olig^ antibody. **c**-**f** Serial sections of a lumbar spinal cord (ventral horn) of the case IV-6 were immunostained with **c**, **e** anti-SOD1^olig^ and **d**, **f** anti-SOD1 (clone 1G2, MBL) antibodies. Sections shown in **c** and **d** or **e** and **f** are serial. Nuclei were also stained by hematoxylin (blue). The bars represent 50 μm

By using anti-SOD1^olig^ antibody, we have further performed immunohistochemical examination on the spinal cords of two *SOD1*-ALS cases with C111Y mutation. Two cases (III-5 and IV-6 reported in [26]; Additional file 1: Table S1) examined here had the disease duration of 1.2 and 4.0 years, respectively. It has been reported that SOD1-positive inclusions are observed at the cytoplasm and neurites of spinal motor neurons in those *SOD1*-ALS cases with C111Y mutation [27]. Unlike G1H mice, those SOD1-positive inclusions in the spinal cord of the case IV-6 were immunostained with anti-SOD1^olig^ antibody (Fig. 3a). In the other *SOD1*-ALS case, III-5, almost no motor neurons were spared due to the intense degeneration of the spinal cord, but sparse immunostaining by anti-SOD1^olig^ antibody was confirmed in the remaining motor neurons of the cervical spinal cord (Fig. 3b). The pathological inclusions in the spinal motor neurons were co-immunostained by anti-SOD1 and anti-SOD1^olig^ antibodies (Fig. 3c-f), and no immunostaining with anti-SOD1^olig^ antibody was confirmed in non-ALS cases (Additional file 5: Figure S4A). Accordingly, anti-SOD1^olig^ antibody can specifically detect the SOD1 species accumulated as pathological inclusions in spinal motor neurons of *SOD1*-ALS patients. This appeared to contradict with the declined immunostaining with anti-SOD1^olig^ antibody in the end stage G1H mice (Fig. 2c and d) but actually support the previous reports describing distinct properties of SOD1-positive inclusions between transgenic mice and human cases [15, 16] (also see Discussion).

These results show that our anti-SOD1^olig^ antibody for the disulfide-crosslinked oligomers was able to detect pathological SOD1 species, but a major concern in the preparation of this antibody is a quite low yield (7.5 mL of 0.2 μg/mL antibody from one immunized rabbit) after several purification procedures. Actually, we have little amounts of anti-SOD1^olig^ antibody left, and constant reproduction of anti-SOD1^olig^ antibody would also be quite difficult due to its polyclonal nature. To deal with those troubles on anti-SOD1^olig^ antibody, we attempted to first determine the epitope of anti-SOD1^olig^ antibody and then produce another oligomer-specific antibody by immunizing rabbits with the peptide covering that epitope.

### Anti-SOD1^olig^ antibody recognizes interior of the SOD1 folded structure

**Fig. 4:**
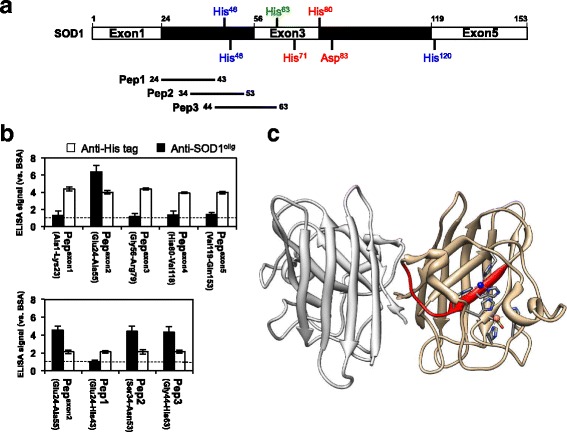
Anti-SOD1^olig^ antibody reacts with the interior of SOD1 structure. **a** The translated products of five exons in SOD1 and the three peptides, Pep 1, 2, and 3, are shown in schematic representation of the SOD1 primary structure. The ligands for copper and zinc ions are also shown and colored blue and red, respectively. His 63 (colored green) is a ligand for bridging both copper and zinc ions. **b** Identification of the epitope for anti-SOD1^olig^ antibody (filled bars) was performed by indirect ELISA using the dissected peptides of SOD1 shown in (**a**). The peptides were prepared as a fusion protein with an N-terminal 6x His tagged GST. Almost equal amounts of peptides were examined, which was confirmed by ELISA using anti-His tag antibody (sc-8036, Santa Cruz Biotechnology) (open bars). The ELISA signal was represented as a ratio against that obtained using BSA. **c** The region covering the epitope of anti-SOD1^olig^ antibody (Gly44 - Asn53) is shown red in the crystal structure of SOD1 (PDB ID: 2C9V). Copper (blue) and zinc (orange) ions are also indicated with the ligands

The epitope mapping of anti-SOD1^olig^ antibody was performed by ELISA using five peptides, Pep^exon1–5^, each of which corresponds to a translated product of five exons in a *SOD1* gene (Fig. 4a). As shown in the upper panel of Fig. 4b, ELISA signals of anti-SOD1^olig^ antibody were observed exclusively in Pep^exon2^, suggesting that the antibody recognizes the region between Glu 24 and Ala 55 in SOD1. To further narrow down the epitope region recognized by anti-SOD1^olig^ antibody, Pep^exon2^ was dissected into three peptides, Pep1 – 3 (Fig. 4a), and again examined by ELISA. Pep2 and 3 but not Pep1 gave rise to ELISA signals (the lower panel of Fig. 4b), indicating that anti-SOD1^olig^ antibody recognized the region overlapped between Pep2 and Pep3, *i.e.* from Gly 44 to Asn 53. Quite interestingly, the epitope (Gly 44 – Asn 53) is buried in the folded structure of SOD1 (Fig. 4c), which is consistent with almost no reactivity of anti-SOD1^olig^ antibody toward folded SOD1 proteins (Fig. 1b). These results thus show that the buried region from Gly 44 to Asn 53 becomes exposed upon formation of the disulfide-crosslinked SOD1 oligomers.

### Antibody recognizing the structural interior of SOD1 exhibits the specificity to the disulfide-crosslinked oligomers

**Fig. 5:**
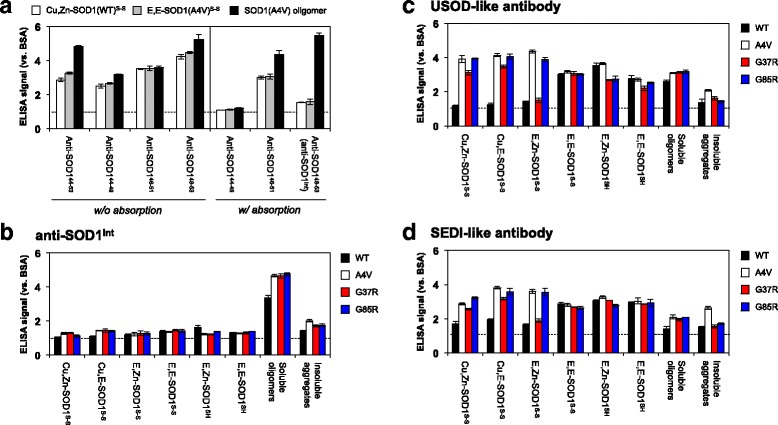
Anti-SOD1^int^ antibody exclusively recognizes soluble disulfide-crosslinked SOD1 oligomers in vitro. **a** The antibodies were tested for their specific reactivities to soluble disulfide-crosslinked oligomers (black filled bars) over Cu,Zn-SOD1(WT)^S-S^ (open bars) and E,E-SOD1(A4V)^S-S^ (gray filled bars) by indirect ELISA. Antisera were either affinity-purified with the corresponding peptides (w/o absorption) or first absorbed with SOD1(WT)^S-S^ and then affinity-purified with the peptides (w/ absorption). Anti-SOD1^48–53^ antibody obtained after the absorption exclusively reacted with soluble disulfide-crosslinked oligomers and called anti-SOD1^int^ antibody. **b**-**d** The reactivities of **b** anti-SOD1^int^, **c** USOD-like, and **d** SEDI-like antibody were examined with indirect ELISA. Several forms of SOD1 (WT, A4V, G37R, G85R) with a distinct metallation/disulfide status, soluble disulfide-crosslinked oligomers and insoluble amyloid-like aggregates were prepared and fixed on an ELISA plate. The ELISA signal was represented as a ratio against that obtained using BSA. Three independent experiments were performed to estimate error bars (standard deviation). Fixation of equal amounts of SOD1 proteins on each well of an ELISA plate was confirmed by ELISA using polyclonal anti-SOD1 antibody (FL-154, Santa Cruz Biotechnology), which is shown in Additional file 6: Figure S5

Polyclonal antibodies were then generated by immunizing a rabbit with the Gly 44 - Asn 53 peptide (Fig. 4c) and affinity-purified using the same peptide. The resultant antibody (anti-SOD1^44–53^) was found to exhibit the increased immunoreactivity to the soluble and disulfide-crosslinked oligomers over the folded Cu,Zn-SOD1(WT)^S-S^ and E,E-SOD1(A4V)^S-S^ (Fig. 5a and Additional file 6: Figure S5A). We then attempted to increase the specificity of the antibody to the oligomers by further affinity-purification with Gly 44 - Glu 49, His 46 - Gly 51, and His 48 - Asn 53 peptides and prepare anti-SOD1^44–49^, anti-SOD1^46–51^, and anti-SOD1^48–53^ antibody, respectively; however, the specificity to the oligomers was not significantly improved in those three antibody fractions (Fig. 5a, w/o absorption). The antisera were, therefore, first absorbed with His-tagged SOD1(WT)^S-S^ on Ni^2+^-affinity resins and then affinity-purified with the peptides (44–49, 46–51, and 48–53) covalently immobilized on Sulfo-Link resins. As shown in Fig. 5a (w/ absorption), such an additional absorption procedure was found to increase the specificity of anti-SOD1^48–53^ antibody to the soluble and disulfide-crosslinked oligomers of A4V-mutant SOD1. We hence call this fraction of anti-SOD1^48–53^ as anti-SOD1^int^ antibody in the following section. The yield of anti-SOD1^int^ antibody was high (*ca*. 150 μg from 1 mL of antisera) enough to conduct its biochemical and immunochemical characterization.

To further check the specificity of our anti-SOD1^int^ antibody in vitro, we prepared various forms of WT and ALS-mutant (A4V, G37R, and G85R) SOD1 proteins including E,E-SOD1^SH^, E,E-SOD1^S-S^, E,Zn-SOD1^SH^, E,Zn-SOD1^S-S^, Cu,E-SOD1^S-S^, Cu,Zn-SOD1^S-S^, and soluble oligomers with disulfide crosslinks (see Fig. 1a). Insoluble and amyloid-like SOD1 aggregates were also prepared by shaking E,E-SOD1^SH^ with 1,200 rpm at 37 °C [9]. Among those, only the soluble and disulfide-crosslinked oligomers but none of the others were recognized by anti-SOD1^int^ antibody (Fig. 5b and Additional file 6: Figure S5B). Based upon these results in vitro, therefore, anti-SOD1^int^ antibody can detect the disulfide-crosslinked SOD1 oligomers, in which the protein interior is significantly exposed to the solvent.

Actually, there are several precedents of the antibodies recognizing the protein interior of SOD1, which include USOD and SEDI polyclonal antibodies raised against the peptides, GG-L^42^HGFHVH^48^-GG and GG-R^143^LACGVIGI^151^-GG, respectively (also see Discussion) [16, 28]. Unfortunately, canonical USOD and SEDI antibodies reported by Chakrabartty and co-workers were not available, but the polyclonal antibodies raised against the same peptides as above were commercially available. We thus characterized those commercially available “USOD-like” and “SEDI-like” antibodies; indeed, USOD-like and SEDI-like antibodies were confirmed to specifically recognize Pep^exon2^ and Pep^exon5^, respectively (Additional file 7: Figure S6). As shown in Fig. 5c and d, USOD-like and SEDI-like antibodies exhibited reactivities toward almost all states examined except wild-type SOD1^S-S^ that is fully or partially metallated and would thus be selective to mutant SOD1 proteins. Nonetheless, the recognition specificity of our anti-SOD1^int^ antibody toward the disulfide-crosslinked oligomers was significantly higher than those of USOD/SEDI-like antibodies (Fig. 5b, c and d). These data thus emphasize the unprecedented recognition specificity of our anti-SOD1^int^ antibody toward the disulfide-crosslinked SOD1 oligomers.

### Disulfide-crosslinked SOD1 oligomers as an early pathological species in spinal cords of ALS-model mice

To check the availability/specificity of anti-SOD1^int^ antibody for the detection of pathological SOD1 in vivo, we first examined the immunohistochemical analysis of G1H mice as well as non-transgenic mice. Unfortunately, however, the lumbar spinal cords of non-transgenic mice were immunostained with anti-SOD1^int^ antibody (data not shown). This is in contrast to anti-SOD1^olig^ antibody showing no immunostaining in non-transgenic mice (Additional file 3: Figure S2A and B). Those two antibodies hence appear to have distinct specificities in the immunohistochemical examination on mouse tissues. Nonetheless, anti-SOD1^int^ as well as anti-SOD1^olig^ antibody can exclusively recognize disulfide-crosslinked SOD1 oligomers in vitro in ELISA (Fig. 1b and 5b). We thus further examined homogenates from model mice for disulfide-crosslinked SOD1 oligomers by sandwich ELISA with anti-SOD1^int^ antibody.

**Fig. 6:**
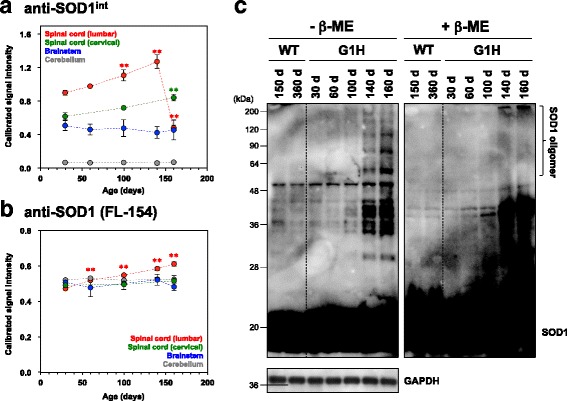
Anti-SOD1^int^ antibody specifically detects pathological SOD1 in spinal cords of ALS-model mice. **a**, **b** SOD1 species recognized by **a** anti-SOD1^int^ and **b** anti-SOD1 (FL-154, Santa Cruz Biotechnology) antibody were quantified in the soluble fraction of the homogenates of lumbar spinal cord (red), cervical spinal cord (green), brainstem (blue), and cerebellum (gray) of G1H mice by sandwich ELISA. Three independent mouse samples at 30, 60, 100, 140, and 160 days of age were examined, and the averages were shown with error bars (standard deviation). ** (red and green) represents the *P* value less than 0.01 versus the data on lumbar and cervical spinal cords at 30 days of age, respectively. **c** Soluble disulfide-crosslinked SOD1 oligomers in mice were examined by Western blotting. Lumbar spinal cords of WT and G1H mice were homogenized in the presence of 100 mM iodoacetamide and 1% NP-40 and centrifuged at 20,000 x *g* for 30 min so as to prepare soluble supernatant. In the presence and absence of the reducing reagent, β-ME, the supernatant was then separated in a polyacrylamide gel by SDS-PAGE and probed by Western blot using anti-SOD1 antibody (FL-154, Santa Cruz Biotechnology). GAPDH was used as a protein loading control for Western blot

Briefly, anti-SOD1^int^ antibody was first fixed on the surface of an ELISA plate and incubated with soluble fractions of tissue homogenates. The SOD1 species captured by the antibody were then detected with polyclonal anti-SOD1 antibody followed by the corresponding secondary antibody. Significant ELISA signals were observed in the lumbar spinal cords of G1H mice from 30 to 140 days of age (Fig. 6a, red circles) but not in those of non-transgenic mice (Additional file 8: Figure S7A). Compared to G1H mice, moreover, asymptomatic mice overexpressing wild-type human SOD1 (WT mice) contained larger amounts of total soluble SOD1 proteins but exhibited significantly weaker signals with anti-SOD1^int^ antibody (Additional file 8: Figure S7A and B). We also observed that the signals with anti-SOD1^int^ antibody disappeared upon pre-treatment of the spinal cord samples of G1H mice with a reductant, dithiothreitol (DTT) (Additional file 8: Figure S7C and D). These results thus suggest that anti-SOD1^int^ antibody detects disulfide-crosslinked SOD1 oligomers not only in in vitro protein samples but also in the model mice in vivo.

It should also be noted in Fig. 6a (red circles) that the ELISA signals from the lumbar spinal cords increase from 30 to 140 days of age but significantly drop from 140 to 160 days of age (*P* < 0.01). The reduction in the ELISA signals at 160 days of age was not described by the changes in total amounts of soluble SOD1 in the lumbar spinal cord (Fig. 6b, red circles). This is consistent with the decreased anti-SOD1^olig^ immunostaining of the lumbar spinal cords of G1H mice at 140 and 160 days of age (Fig. 2c and d, Additional file 4: Figure S3C and D), while slightly different age-dependency in immunochemical response between anti-SOD1^int^ and anti-SOD1^olig^ antibodies would reflect their distinct immunological properties. As described later, however, the ELISA signals with anti-SOD1^int^ antibody did not decrease in the other mouse model. The signal reduction in the disease end-stage may thus be a phenomenon specific to the lumbar spinal cord of G1H mice, but an exact reason for this remains obscure. Instead, we would like to emphasize that anti-SOD1^int^ antibody can detect conformationally abnormal SOD1 species in model mice with sandwich ELISA.

To test if SOD1s were oligomerized with disulfide bonds in lumbar spinal cords of ALS-model mice, soluble fractions of the lumbar spinal cord homogenates of G1H mice were separated by non-reducing SDS-PAGE and analyzed by Western blotting. Soluble and disulfide-crosslinked SOD1 oligomers in vitro can be characterized by the reductant-sensitive smears in the high molecular weight region in SDS-PAGE gels (Additional file 2: Figure S1). As shown in Fig. 6c (left panel), smears in the high molecular weight region (>50 kDa) were evident, albeit weak intensity, as early as 60 days of age in G1H mice but not in WT mice (150 and 360 days) and non-transgenic mice (100 days; data not shown). Also importantly, those smears in G1H mice at 60 and 100 days of age disappeared when the soluble fractions were treated with β-mercaptoethanol (β-ME) prior to their loading on an SDS-PAGE gel (Fig. 6c, right panel), supporting the formation of disulfide-crosslinked SOD1 oligomers in the ALS-model mice even before the disease onset.

After the disease onset (at 140 and 160 days of age), in contrast, the reductant-sensitive SOD1 species in lumbar spinal cords of G1H mice were observed as more distinct bands in the high molecular weight region (>50 kDa, Fig. 6c). Also, even in the presence of β-ME, some SOD1-positive species were stuck on top of the separating gel. Therefore, we suppose different molecular properties of SOD1 oligomers between pre- and post-symptomatic stages of G1H mice, which might describe significant reduction of the SOD1 species immunoreactive to anti-SOD1^int^ antibody at 160 days of age (Fig. 6a, red circles). Taken together, we speculate that anti-SOD1^int^ antibody specifically detects the disulfide-crosslinked oligomers formed in the lumbar spinal cords of G1H mice from their pre-symptomatic stages.

We also tested the tissue-specificity in the formation of the disulfide-crosslinked oligomers; soluble supernatants from the homogenates of cervical spinal cord, brainstem and cerebellum of G1H mice were examined by sandwich ELISA using anti-SOD1^int^ antibody. In ALS cases, the lumbar spinal cord is mainly affected, but the other regions of brains and spinal cords have also been shown to be involved in the pathology [29]. In G1H mice, the lumbar spinal cord is the most severely damaged, and the changes occur later in the cervical spinal cords [30]. Some pathological changes are reported in the brainstem [31], but the cerebellum is relatively spared [32]. As shown in Fig. 6a, the ELISA signal intensities were significantly weaker in cervical spinal cord, brainstem, and cerebellum than those of lumbar spinal cord (*P* < 0.01 within the same age group, except at 160 days of age); in particular, almost no ELISA signals were observed in cerebellum. We confirmed similar levels of total SOD1 proteins among all of those tissues (Fig. 6b). Also, no obvious smears in the high molecular weight region were observed in the Western blots of soluble fractions of the cerebellum, while the brainstem lysates of G1H mice exhibited reductant-sensitive smears at 160 days of age, albeit with weak intensities (Additional file 9: Figure S8A and B). While toxic SOD1 species might appear everywhere but only afflict the spinal cord due to its vulnerability, amounts of SOD1 species probed with anti-SOD1^int^ antibody were well correlated with the intensity of high-molecular-weight smears in the Western blots and also the severity of the damages in tissues (lumbar spinal cord > cervical spinal cord > brainstem > cerebellum) of G1H mice.

We have also examined the ALS model mice expressing human SOD1 with another mutation, G37R (loxG37R mice) [20]. Compared to G1H mice, the expression level of mutant SOD1 is lower, and the disease progression is slower in loxG37R mice (the disease onset: ~350 days of age). The sandwich ELISA showed the age-dependent increase of the anti-SOD1^int^-positive SOD1 species in lumbar and cervical spinal cords but not in cerebellum of loxG37R mice (Additional file 10: Figure S9A), while amounts of the total soluble SOD1 remained almost constant during aging in loxG37R mice (Additional file 10: Figure S9B). When the spinal cord samples from loxG37R mice were pre-treated with DTT, the ELISA signals with anti-SOD1^int^ antibody disappeared (data not shown). Furthermore, Western blotting analysis on loxG37R mice revealed the reductant-sensitive smears in lumbar spinal cord (Additional file 10: Figure S9C), albeit with significantly weaker intensities compared to those of G1H mice, but not in cerebellum (Additional file 9: Figure S8C). Taken together, these results suggest the formation of the soluble disulfide-crosslinked SOD1 oligomers as pathological changes also in loxG37R mice.

### Anti-SOD1^int^ antibody detects pathological SOD1 in *SOD1*-ALS cases

**Fig. 7:**
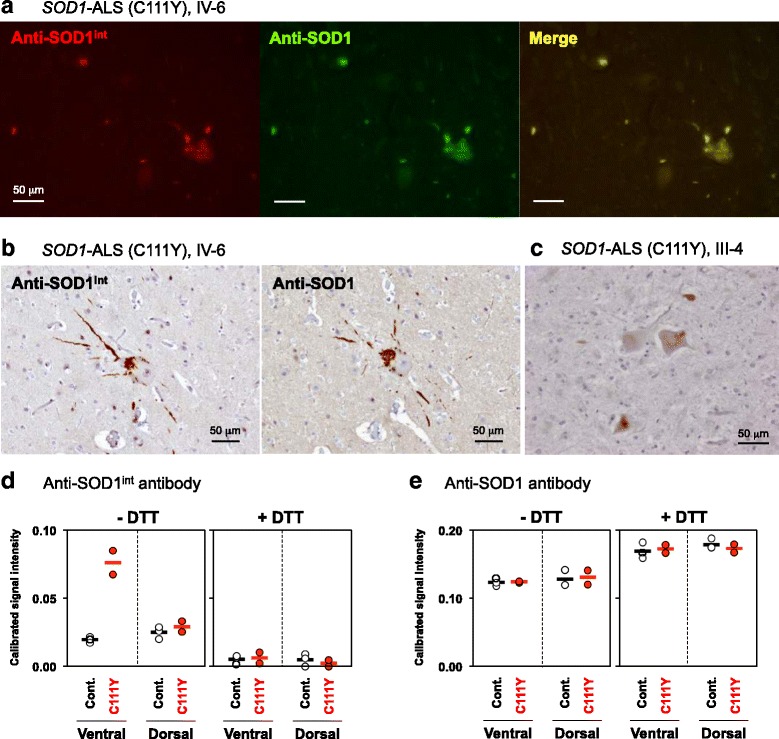
Immunochemical detection of pathological SOD1 species in the *SOD1*-ALS cases with anti-SOD1^int^ antibody. **a** Double immunofluorescence staining of the lumbar spinal cord section of the *SOD1*-ALS case with C111Y mutation (the case IV-6). The section was stained with rabbit anti-SOD1^int^ and mouse anti-SOD1 (clone 1G2, MBL) antibodies followed by Cy3-modified anti-rabbit (red) and FITC-modified anti-mouse (green) secondary antibodies, respectively. A merged image (yellow) was also shown. **b** Serial sections of the primary motor cortex of the *SOD1*-ALS case (the case IV-6) were immunostained with (left) anti-SOD1^int^ and (right) anti-SOD1 (clone 1G2, MBL) antibodies and visualized with DAB staining. **c** The lumbar spinal cord (ventral horn) of the case III-4, which exhibited exceptionally long disease duration (~69 years), was immunostained with anti-SOD1^int^ antibody. **d**, **e** SOD1 species recognized by **d** anti-SOD1^int^ and **e** anti-SOD1 (FL-154, Santa Cruz) antibodies were quantified in the soluble fraction of the homogenates of ventral and dorsal horn regions of thoractic spinal cord of human samples by sandwich ELISA. The sandwich ELISA was performed in the absence (−DTT) or presence (+DTT) of pre-treatment of the samples with 10 mM DTT. The *SOD1*-ALS cases III-4 and IV-6 and three non-ALS controls (Additional file 1: Table S1) were examined. The averages were shown as bars

To test the immunoreactivity of our anti-SOD1^int^ antibody in human cases, the double immunofluorescence staining with anti-SOD1^int^ and anti-SOD1 antibodies was performed on the ventral horn of the lumbar spinal cord section of the *SOD1*-ALS patients with C111Y mutation (the case IV-6 in [26]; Additional file 1: Table S1). As shown in Fig. 7a, abnormally accumulated SOD1 proteins in spinal motor neurons were immunostained by anti-SOD1^int^ antibody. Using serial sections of the primary motor cortex (Fig. 7b), furthermore, the abnormally accumulated SOD1 in cytoplasm and neurites of a Betz cell were also immunostained by anti-SOD1^int^ antibody. In another *SOD1*-ALS case with C111Y mutation (the case III-4 in [26]; Additional file 1: Table S1), the disease duration was exceptionally long (69 years), but the motor neurons in the ventral horn of the lumbar spinal cord exhibited immunoreactivity to anti-SOD1^int^ antibody (Fig. 7c). Again, no immunoreactivity with anti-SOD1^int^ antibody was observed in the spinal cord/primary motor cortex of the sporadic ALS cases with TDP-43 pathologies and also of the non-ALS cases (Additional file 5: Figure S4B-D).

To reduce the chance of potential conformational changes of SOD1 during the preparation of sections and their immunostaining procedures, we prepared soluble fractions by centrifugation of the homogenates from either ventral or dorsal horn in the thoractic spinal cords and then examined those by sandwich ELISA using anti-SOD1^int^ antibody. ALS associates with degeneration of motor neurons in the ventral horn with less involvement of sensory neurons in the dorsal horn of the spinal cord [33]. As shown in Fig. 7d, the ventral horn of the *SOD1*-ALS patients with C111Y mutation (cases III-4 and IV-6; Additional file 1: Table S1) showed higher signal intensities of anti-SOD1^int^ antibody than those of non-ALS controls (three cases). In contrast, weak ELISA signals of anti-SOD1^int^ antibody were detected in the dorsal horn with almost no difference between the controls and the ALS patients (Fig. 7d). Also importantly, the ELISA signals obtained by using anti-SOD1^int^ antibody disappeared upon pre-treatment of the samples with a reductant, DTT, suggesting the involvement of disulfide-crosslinks in the pathological SOD1 species (Fig. 7d). The amounts of total soluble SOD1 proteins in the ventral and dorsal horns were not different between the controls and the ALS patients (Fig. 7e). Collectively, our anti-SOD1^int^ antibody is considered to detect the disulfide-crosslinked SOD1 oligomers as pathological species in *SOD1*-ALS patients.

## Discussion

*SOD1*-ALS cases are characterized mainly by abnormal accumulation of mutant SOD1 proteins in motor neurons of the affected spinal cords [5], while the pathological involvement of the other types of neurons and glia cells has been reported [20, 29, 34, 35]. The conformational stability of SOD1 is significantly compromised by most of the mutations [36], which triggers the formation of soluble oligomers and insoluble aggregates of SOD1 in vitro. In this study, we successfully prepared anti-SOD1^olig/int^ antibodies exclusively recognizing the disulfide-crosslinked SOD1 oligomers in vitro and then found that those antibodies detect the pathological SOD1 species in spinal cords of the *SOD1*-ALS patients (C111Y) as well as transgenic model mice (G1H and loxG37R mice).

**Fig. 8:**
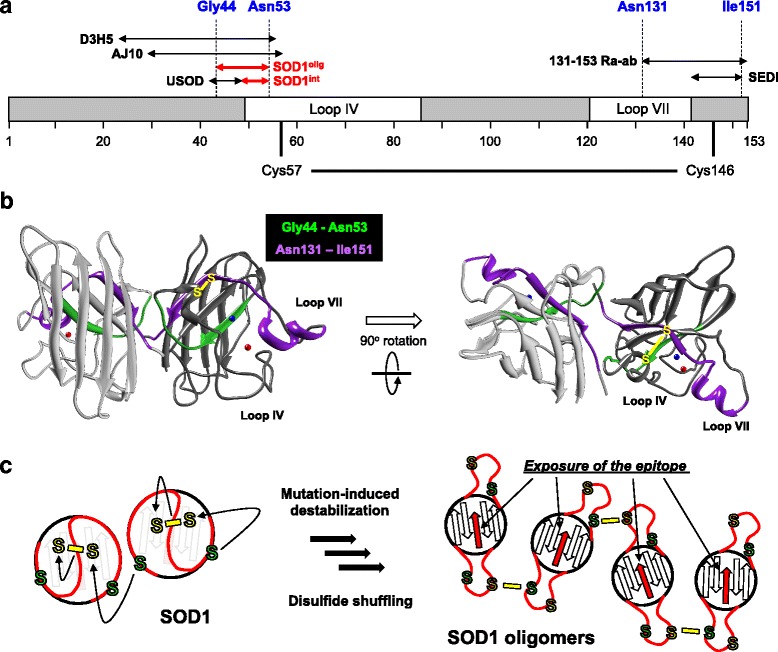
A disulfide shuffling mechanism describes exposure of pathological epitope in SOD1. (**a**) Schematic representation of the epitopes of the antibodies that exclusively detect the pathological inclusions in *SOD1*-ALS cases. (**b**) The regions of the overlapped epitopes among antibodies specific to pathological SOD1 (Gly 44 - Asn 53, green; Asn 131 - Ile 151, purple) are mapped on the crystal structure of the native SOD1 protein (PDB ID: 2C9V). Together with the intramolecular disulfide bond (Cys 57 - Cys 146) in yellow, copper and zinc ions are also shown as blue and red ball models, respectively. (**c**) Schematic representation of the epitope exposure through the disulfide shuffling mechanism. Cys 57 and 146, which form the canonical disulfide bond, are shown as “S” in yellow, while the other two free Cys residues, Cys 6 and 111, as S in green. Red curves represent loops IV and VII. Mutation-induced conformational disorder of SOD1 allows the free Cys (green S) to nucleophilically attack and then shuffle the Cys 57 - Cys 146 disulfide bond (yellow). The disulfide shuffling within and between SOD1 molecules is considered to peel the loops (red) off from the structural core of SOD1 and thereby expose the epitope (arrows in red) for our antibodies

Several mechanisms for the formation of disulfide-crosslinked SOD1 oligomers have been proposed in vitro [7, 8, 37]. Our epitope analysis of anti-SOD1^olig/int^ antibodies has further revealed the conformation of disulfide-crosslinked SOD1 oligomers, in which the regions usually buried in the native SOD1 are exposed. More precisely, the epitopes of our antibodies (Gly 44 - Asn 53) were found to include the protein interior (Gly 44 - Glu 49) and the dimer interface (Phe 50 - Asn 53) in SOD1. The protein interior and the dimer interface have been noted as targets for the design of antibodies specifically recognizing misfolded SOD1 proteins [38]. For example, the polyclonal antibodies, USOD [16] and AJ10 [39], were raised against the region covering the protein interior of the natively folded SOD1 (Leu 42 - His 48 and Val 29 - Cys 57, respectively) (Fig. 8a), and both antibodies have been shown to immunostain the pathological inclusions in the spinal motor neurons of *SOD1*-ALS patients. Also, the monoclonal antibodies, C4F6 and D3H5, have been reported to recognize the conformational epitope buried in the SOD1 native conformation [38, 40–42] and detect pathological inclusions in *SOD1*-ALS patients [43, 44]. Because SOD1 forms a very tight homodimer (*K*_d_ ~ 0.1 nM) [45], furthermore, the dimer interface in the natively folded conformation is also buried. The polyclonal antibodies called SEDI and 131–153 Ra-ab were raised against the peptides containing the dimer-interface region (SEDI, Arg 143 - Ile 151; 131–153 Ra-ab, Asn 131 - Gln 153; Fig. 8a and b) [28, 46] and again exclusively immunostained the inclusions in the affected spinal motor neurons of *SOD1*-ALS patients [16, 28, 47–50]. In the pathological conditions, therefore, SOD1 is supposed to misfold into a conformation with the exposed structural interior and the disrupted interface for dimerization.

Depending upon experimental conditions in vitro and in vivo, nonetheless, SOD1 is known to misfold in distinct pathways [8–10, 51–54]. Therefore, we could not exclude the possibility that our antibodies detect certain misfolded species other than the disulfide-crosslinked oligomer. It has been known that several misfolded conformations of SOD1 can be reproduced in vitro simply by metal dissociation and/or disulfide reduction. Actually, we have shown that reduction of the disulfide bond drastically increases fluctuation of the loops IV and VII (Fig. 8b), temporarily “peels” those loops off from the β-barrel scaffold, and thus potentially exposes the epitope of anti-SOD1^int^ antibody [55]. As shown in Fig. 5b, however, disulfide-reduced and/or demetallated forms of SOD1 were not recognized by our anti-SOD1^int^ antibody. Therefore, the reversible conformational fluctuation increased by disulfide reduction/demetallation is probably not sufficient to allow the antibody to access the epitope buried inside the protein. The disulfide-reduced and demetallated SOD1 has been shown to irreversibly form the amyloid-like aggregates [56], which were again not recognized by anti-SOD1^int^ antibody (Fig. 5b). We further examined ELISA to test the reactivities of anti-SOD1^int^ antibody toward E,E-SOD1^S-S^ and E,E-SOD1^SH^ that were misfolded/unfolded with either guanidine hydrochloride (6 M) or acidic buffer (pH 3.0) in vitro, but no signals were observed (Additional file 11: Figure S10A). In sharp contrast, USOD-like and SEDI-like antibodies were found to react with various non-native forms of SOD1 proteins (Fig. 5c and d, Additional file 11: Figure S10B, C, and D). Taken together, those extensive tests reveal quite high recognition specificity of our anti-SOD1^int^ antibody toward the disulfide-crosslinked SOD1 oligomer.

Anti-SOD1^int^ antibody detected pathological SOD1 species in vivo, and also, the reductant-sensitive smears of SOD1 were observed in the Western blots of the affected tissues of model mice (Fig. 6, Additional file 10: Figure S9). We hence speculate that the disulfide-crosslinked SOD1 oligomer is involved in the pathology of *SOD1*-ALS. Actually, disulfide-crosslinked oligomers of SOD1 have been reproduced in cultured cells [18, 57]. While the reducing environment of the cytoplasm might be unfavorable for crosslinking proteins *via* disulfide bonds, our preliminary in vitro experiments confirmed the formation of disulfide-crosslinked SOD1 oligomers in the presence of 5 mM reduced glutathione with 0.5 mM oxidized glutathione (data not shown), which is a feasible redox condition of the cytoplasm [58]. In our proposed mechanism for the formation of SOD1 oligomers [8], the disulfide bond is not newly introduced but rather shuffled among the Cys residues in SOD1 (Fig. 8c). More specifically, the disulfide shuffling will break the canonical Cys 57 - Cys 146 disulfide bond and detain SOD1 in the misfolded conformation where the loops IV and VII are peeled off from the β-barrel scaffold with the persistent exposure of the epitope region (Fig. 8c). Such a disulfide shuffling may be more robust against reducing environment than *de novo* formation of disulfide bonds.

We have also shown here that immunoreactivities of our anti-SOD1^olig/int^ antibodies are quite exclusive to the human *SOD1*-ALS cases as well as the transgenic mice (G1H and loxG37R mice) but not to the controls without *SOD1* mutations. Their immunoreactivities were, furthermore, well correlated with the severity of degeneration in the model mice (lumbar spinal cord > cervical spinal cord > brainstem > cerebellum; Fig. 6a, Additional file 10: Figure S9A) and in the *SOD1*-ALS cases (ventral horn > dorsal horn; Fig. 7d). As described in the Results section, nonetheless, we should note significant reduction in the immunoreactivities of anti-SOD1^olig/int^ antibodies toward the G1H mice in the disease end stage (Figs. 2 and 6a). While such decline will be partly because of the concomitant loss of motor neurons [24], mutant SOD1 is also known to accumulate as amyloid-like aggregates in the spinal cord of the model mice after the appearance of motor symptoms [9, 15]. Given that our anti-SOD1^int^ antibody was not able to react with the amyloid-like SOD1 aggregates in vitro (Fig. 5b), the declined immunoreactivity of anti-SOD1^olig/int^ antibodies at the end-stage might indicate the formation of amyloid-like SOD1 aggregates due to quite high expression of G93A SOD1 proteins in G1H mice.

In contrast, the spinal motor neurons of the *SOD1*-ALS patients in their disease end-stage were immunostained with anti-SOD1^olig/int^ antibodies (Figs. 3 and 7), suggesting distinct properties of pathological SOD1 species between G1H mice and the patients. Because the inclusions in *SOD1*-ALS patients exhibited no reactivity to an amyloid-diagnostic dye, Thioflavin-S [16], the amyloid-like SOD1 aggregates would form only in the end-stage G1H mice but not in human *SOD1*-ALS cases. While it needs to be tested whether the SOD1 species detected by our antibodies was an on-pathway intermediate for the formation of amyloid-like SOD1 aggregates, the pathologies in the autopsied human cases might not proceed into the terminal stage as in the G1H mice at 160 days of age. Compared to the amyloid-like SOD1 aggregates in the end stage of G1H mice, we speculate that SOD1 species detected by anti-SOD1^olig/int^ antibodies in the pre-symptomatic G1H mice have more significance in the pathogenicity of *SOD1*-ALS.

It is also important to note moderate immunoreactivities of anti-SOD1^int^ antibody in the spinal cords of WT mice at 360 days but not at 150 days of age (Additional file 8: Figure S7A). WT mice do not develop severe motor phenotypes but show several neuropathological and symptomatic changes in their advanced age including mitochondrial vacuolization in spinal cords (>210 days), impaired motor performance (>410 days), and motor neuron death (~2 years) [59]. No immunoreactivities of C4F6 and SEDI antibodies were reported in the spinal cords of WT mice at 215 and 100 days of age, respectively [28, 43]. Instead, the immunoreactivity of anti-SOD1^int^ antibody appears to match with the neuropathological changes in WT mice and would hence probe the misfolded SOD1 proteins with toxicity toward motor neurons. Involvement of wild-type SOD1 proteins in the ALS pathogenesis still remains controversial [60], and no immunostaining by anti-SOD1^int^ antibody was confirmed in our sporadic ALS cases without *SOD1* mutations (Additional file 5: Figure S4C and D). Nonetheless, we speculate that SOD1 could become toxic to motor neurons by assuming the anti-SOD1^olig/int^ antibody-positive conformation such as the disulfide-crosslinked oligomers even in the absence of any pathogenic mutations.

## Conclusions

In summary, we successfully prepared the antibodies exclusively recognizing the disulfide-crosslinked SOD1 oligomers in vitro. Pathological SOD1 species in the affected tissues of *SOD1*-ALS patients as well as transgenic mice provided the immunological epitope to those antibodies. While it is possible that the epitope of our antibodies becomes available upon misfolding of SOD1 through some other mechanisms, we propose that the oligomerization *via* shuffling of the disulfide bond has pathological significance in *SOD1*-ALS.

## Additional files

Additional file 1: Table S1. Information on human cases examined in this study. (PDF 49 kb)
Additional file 2: Figure S1. Preparation of soluble disulfide-crosslinked SOD1 oligomers in vitro. E,E-SOD1^S-S^ (100 μM) was incubated in the NNE buffer at 37 °C for five days and then centrifuged at 20,000 x *g* for 10 min. to remove any insoluble materials. The samples were further reacted with 100 mM iodoacetamide in the presence of 2% SDS and analyzed by (left) non-reducing and (right) reducing SDS-PAGE. The electrophoretic mobility of monomeric SOD1(G85R) has been known to be faster than those of the other SOD1 proteins (WT, A4V, and G37R). (PDF 505 kb)
Additional file 3: Figure S2. Specificity of anti-SOD1^olig^ antibody for immunohistochemical examination of mouse spinal cords. The sections of lumbar spinal cords of (A, B) a non-transgenic mouse (C57BL/6) at 200 days of age and (C, D) a G1H mouse at 100 days of age were stained with anti-SOD1^olig^ antibody. For the absorption experiments shown in (C, D), the anti-SOD1^olig^ antibody was pre-absorbed on ice for 5 h with either (C) soluble disulfide-crosslinked SOD1(A4V) oligomers (6.7 μM in monomer base) or (D) E,Zn-SOD1^S-S^ (6.7 μM). Nuclei were also counterstained with hematoxylin (blue). (PDF 265 kb)
Additional file 4: Figure S3. Immunohistochemical examination on lumbar spinal cords of G1H mice with anti-SOD1 antibody. The sections of lumbar spinal cords of G1H mice at (A) 60, (B) 100, (C) 140, and (D) 160 days of age were stained with monoclonal anti-SOD1 (clone 1G2, MBL) antibody. The images in the low magnification are shown in the left panel, where the region enclosed with a broken line is magnified and shown in the right panel. Nuclei were counterstained with hematoxylin (blue). The bar in each panel represents 100 μm (left panel) and 50 μm (right panel). (PDF 273 kb)
Additional file 5: Figure S4. Representative images for immunohistochemical examination of non *SOD1*-ALS cases. Spinal cord sections of (A) non-ALS (C1 in Additional file 1: Table S1), (B) non-ALS (C3 in Additional file 1: Table S1), (C) sporadic ALS (sALS3 in Additional file 1: Table S1), and (D) sporadic ALS (sALS4 in Additional file 1: Table S1) cases were immunostained with either (A) anti-SOD1^olig^ or (B-D) anti-SOD1^int^ antibody. Nuclei were also stained by hematoxylin (blue). The bars represent 50 μm. (PDF 1002 kb)
Additional file 6: Figure S5. Equal fixation of various forms of SOD1 proteins on the ELISA plate. Amounts of SOD1 proteins fixed on the ELISA plates for the experiments in Fig. 5 were quantified by indirect ELISA with polyclonal anti-SOD1 antibody (FL-154, Santa Cruz Biotechnology). Amounts of SOD1 in the plates for experiments in Fig. 5a were shown in the panel (A), while those in Fig. 5b, c, and d were in the panel (B). The ELISA signal was represented as a ratio against that obtained using BSA. Three independent experiments were performed to estimate error bars (standard deviation). No statistically significant difference in the ELISA signal was confirmed among the in vitro samples examined. (PDF 52 kb)
Additional file 7: Figure S6. Epitope analysis on USOD-like and SEDI-like antibodies by indirect ELISA. The peptides were prepared as a fusion protein with an N-terminal 6x His tagged GST and adsorbed on an ELISA plate. SOD1 species recognized by (A) USOD-like and (B) SEDI-like antibodies were quantified as ELISA signals that were represented as a ratio against those of BSA. Almost equal amounts of peptides were adsorbed on the plate, which was confirmed by an ELISA using anti-GST antibody (C). Three independent experiments were performed to estimate error bars (standard deviation). (PDF 29 kb)
Additional file 8: Figure S7. Anti-SOD1^int^ antibody specifically detects pathological SOD1 in spinal cords of ALS-model mice. (A, B) SOD1 species recognized by (A) anti-SOD1^int^ and (B) anti-SOD1 (FL-154, Santa Cruz Biotechnology) antibody were quantified in the soluble fraction of the homogenates of lumbar spinal cords of non-transgenic (nonTG), WT, and G1H mice by sandwich ELISA. The data on G1H mice at 30 days of age are the same with those in Fig. 6a and b and shown here again for comparison. (C, D) Sandwich ELISA with (C) anti-SOD1^int^ and (D) anti-SOD1 (FL-154, Santa Cruz Biotechnology) antibody was examined by using the soluble fraction of the homogenates of lumbar spinal cords of G1H mice. The samples were pre-treated with 10 mM DTT for reducing disulfide bonds. In all panels, three independent mouse samples were examined to estimate error bars (standard deviation), and the statistical analysis has been performed to obtain the *P* values indicated in the figures. (PDF 47 kb)
Additional file 9: Figure S8. Examination of soluble SOD1 disulfide-crosslinked oligomers in cerebellum and brainstem of ALS-model mice. (A) Cerebellum and (B) brainstem of non-transgenic (NTG) and G1H mice and (C) cerebellum of loxG37R mice were homogenized in the presence of 100 mM iodoacetamide and 1% NP-40 and centrifuged at 20,000 x *g* for 30 min so as to prepare soluble supernatant. The supernatant was then separated in a polyacrylamide gel by non-reducing SDS-PAGE and probed by Western blot using anti-SOD1 antibody (FL-154, Santa Cruz Biotechnology). For comparison, the soluble fraction of the lumbar spinal cord homogenates of G1H mice at 160 days of age was also loaded on the same gel. GAPDH was used as a protein loading control for Western blot. (PDF 812 kb)
Additional file 10: Figure S9. Anti-SOD1^int^ antibody specifically detects pathological SOD1 in spinal cords of loxG37R mice. (A, B) SOD1 species recognized by (A) anti-SOD1^int^ and (B) anti-SOD1 (FL-154, Santa Cruz Biotechnology) antibody were quantified in the soluble fraction of the homogenates of lumbar spinal cord (red), cervical spinal cord (green), brainstem (blue), and cerebellum (gray) of loxG37R mice by sandwich ELISA. Ages of the mouse samples examined are as follows; 100 (three independent mice), 174 (two independent mice), 188, 374 (three independent mice), 386, 392, and 445 days of age. The data were divided into two groups before and after the onset of the disease (350 days of age) and statistically analyzed by two-tailed student’s *t* test. The difference in the signal intensity before and after the disease onset was statistically significant in lumbar and cervical spinal cords (**: *P* < 0.01). (C) Soluble disulfide-crosslinked SOD1 oligomers in loxG37R mice were examined by Western blotting. Lumbar spinal cords of loxG37R and G1H mice at indicated days of ages were homogenized in the presence of 100 mM iodoacetamide and 1% NP-40 and centrifuged at 20,000 x *g* for 30 min so as to prepare soluble supernatant. In the presence and absence of the reducing reagent, β-ME, the supernatant was then separated in a polyacrylamide gel by SDS-PAGE and probed by Western blot using anti-SOD1 antibody (FL-154, Santa Cruz Biotechnology). GAPDH was used as a protein loading control for Western blot. (PDF 870 kb)
Additional file 11: Figure S10. Reactivity of the antibodies toward chemically misfolded/unfolded forms of SOD1. E,E-SOD1^SH^ and E,E-SOD1^S-S^ (100 μM; WT, A4V, G37R, and G85R) were first incubated at room temperature for two hours either in 50 mM Tris/100 mM NaCl/5 mM EDTA/6 M guanidine hydrochloride (GdnHCl) at pH 7.4 or in 50 mM sodium acetate buffer at pH 3.0 and then fixed on an ELISA plate. ELISA was performed using (A) anti-SOD1^int^, (B) USOD-like, (C) SEDI-like, and (D) anti-SOD1 (FL-154, Santa Cruz Biotechnology) antibodies. The ELISA signal was represented as a ratio against that obtained using BSA. Three independent experiments were performed to estimate error bars (standard deviation). (PDF 49 kb)

## Abbreviations

ALS: Amyotrophic lateral sclerosis
G1H mouse: A transgenic mouse carrying human *SOD1* gene with G93A mutation
loxG37R mouse: A transgenic mouse carrying SOD1 gene with G37R mutation
SOD1: Cu/Zn-superoxide dismutase
*SOD1*-ALS: ALS with mutations in SOD1 gene
WT mouse: A transgenic mouse carrying human wild-type *SOD1* gene.
