# Supplementary material for: Immunochemical characterization on pathological oligomers of mutant Cu/Zn-superoxide dismutase in amyotrophic lateral sclerosis
Source: Mol Neurodegener. 2017 Jan 5;12:2. doi: 10.1186/s13024-016-0145-9 (PMC5216565; doi:10.1186/s13024-016-0145-9)
Supplement: Additional file 1: Table S1. — Information on human cases examined in this study. (PDF 49 kb) [file 13024_2016_145_MOESM1_ESM.pdf]

Table S1 Information on human cases examined in this study

Case	Diagnosis	Mutation	Disease duration (years)	First neurological symptom
III-4	Familial ALS	SOD1 C111Y	69	Weakness in the upper limb
III-5	Familial ALS	SOD1 C111Y	1.2	Weakness in the right leg
IV-6	Familial ALS	SOD1 C111Y	4	Weakness in the right hand
sALS1	Sporadic ALS	N/A	1.8	Weakness in the lower limb
sALS2	Sporadic ALS	N/A	2.8	Involuntary bend in the left upper limb
sALS3	Sporadic ALS	N/A	3.7	Weakness in the right upper limb
sALS4	Sporadic ALS	N/A	2.6	Weakness in the right upper limb
C1	Multiple cerebral infarction	N/A	N/A	N/A
C2	AL amyloidosis	N/A	N/A	N/A
C3	Myotonic dystrophy	N/A	N/A	N/A
