# Supplementary figures and images for: Immunochemical characterization on pathological oligomers of mutant Cu/Zn-superoxide dismutase in amyotrophic lateral sclerosis
Source: Mol Neurodegener. 2017 Jan 5;12:2. doi: 10.1186/s13024-016-0145-9 (PMC5216565; doi:10.1186/s13024-016-0145-9)

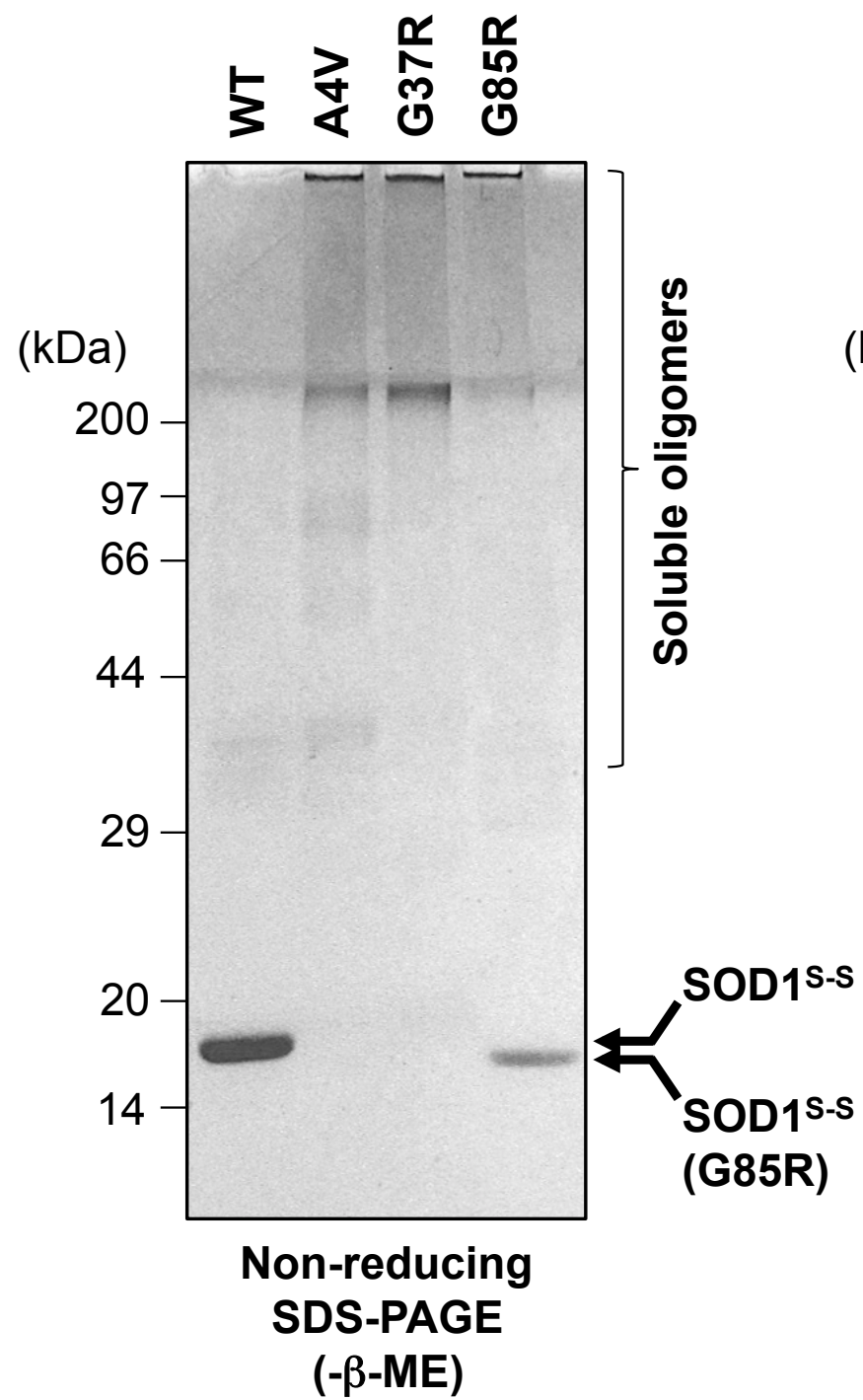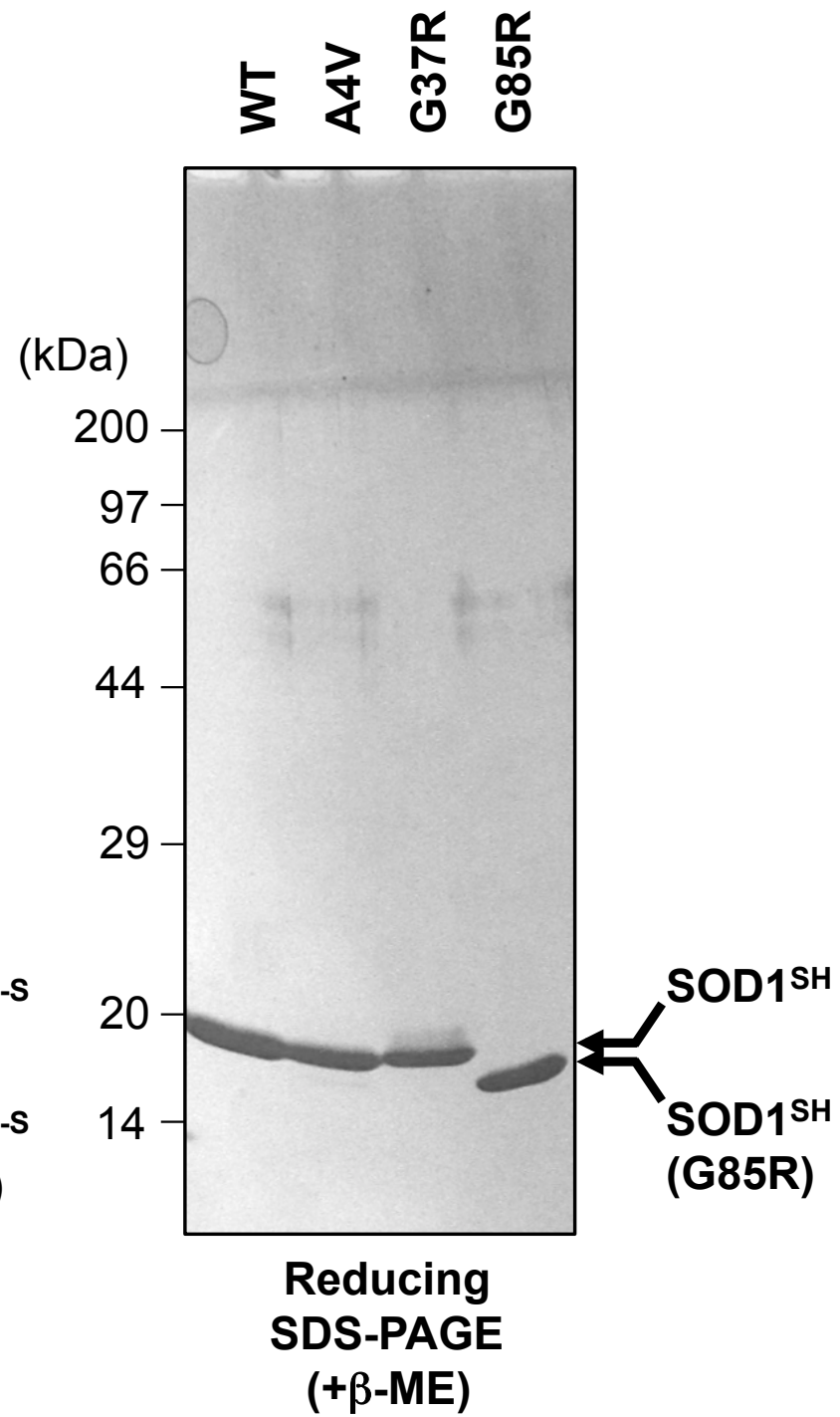

Supplement: Additional file 2: Figure S1. — Preparation of soluble disulfide-crosslinked SOD1 oligomers in vitro. E,E-SOD1S-S (100 μM) was incubated in the NNE buffer at 37 °C for five days and then centrifuged at 20,000 x g for 10 min. to remove any insoluble materials. The samples were further reacted with 100 mM iodoacetamide in the presence of 2% SDS and analyzed by (left) non-reducing and (right) reducing SDS-PAGE. The electrophoretic mobility of monomeric SOD1(G85R) has been known to be faster than those of the other SOD1 proteins (WT, A4V, and G37R). (PDF 505 kb) [file 13024_2016_145_MOESM2_ESM.pdf]

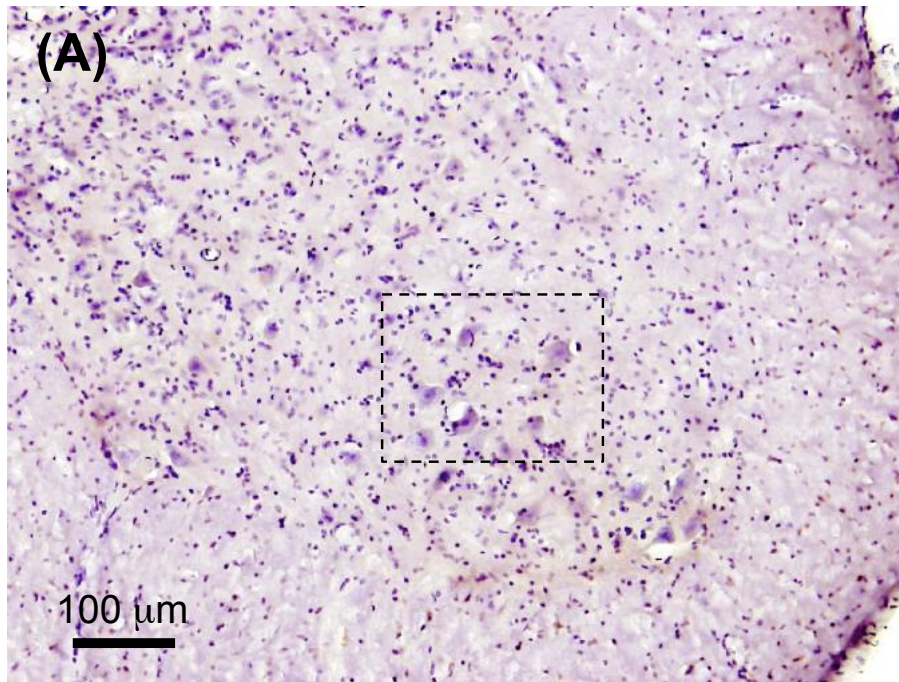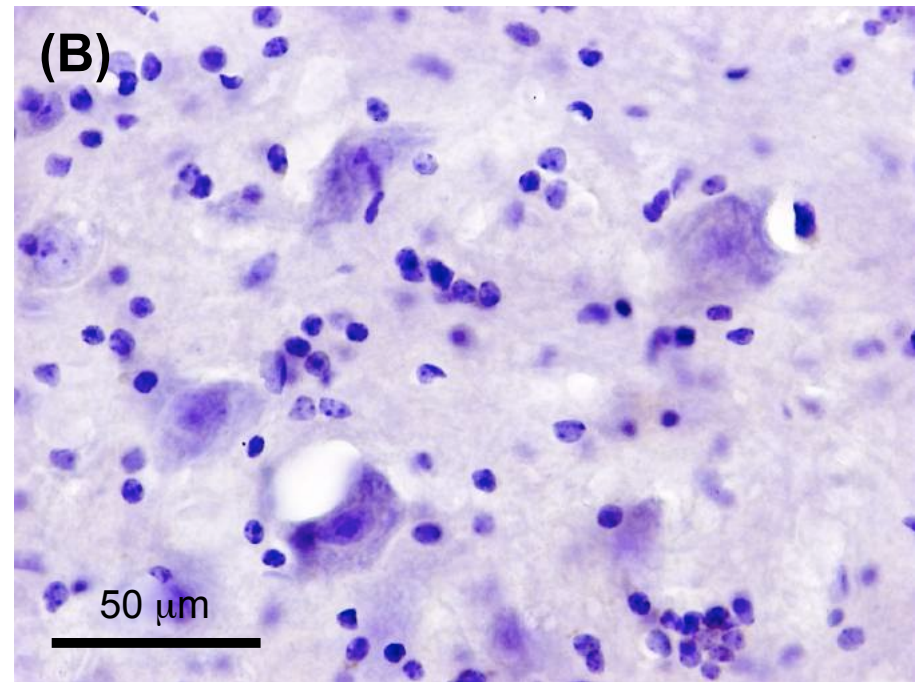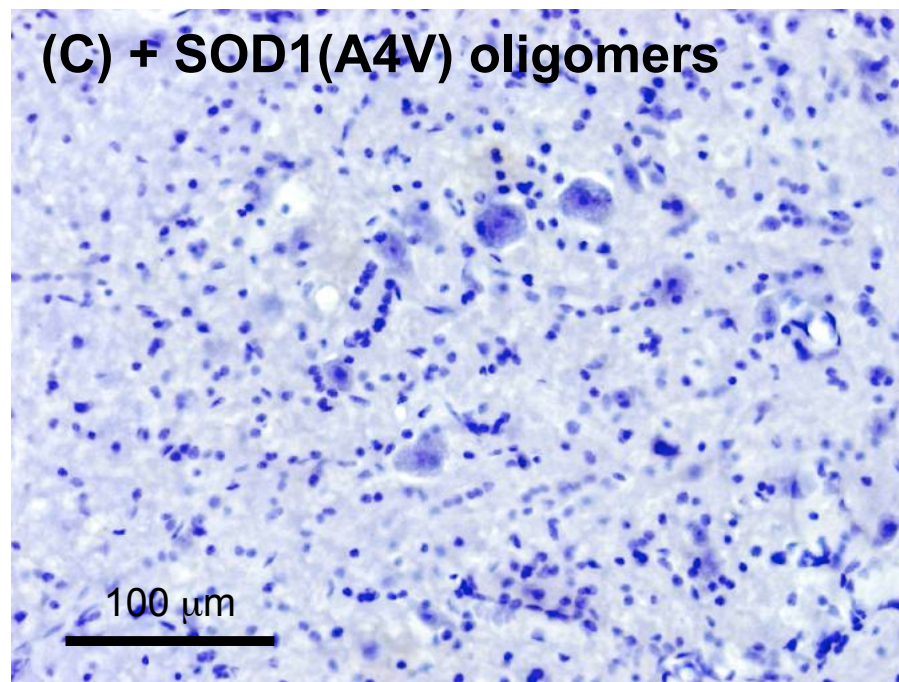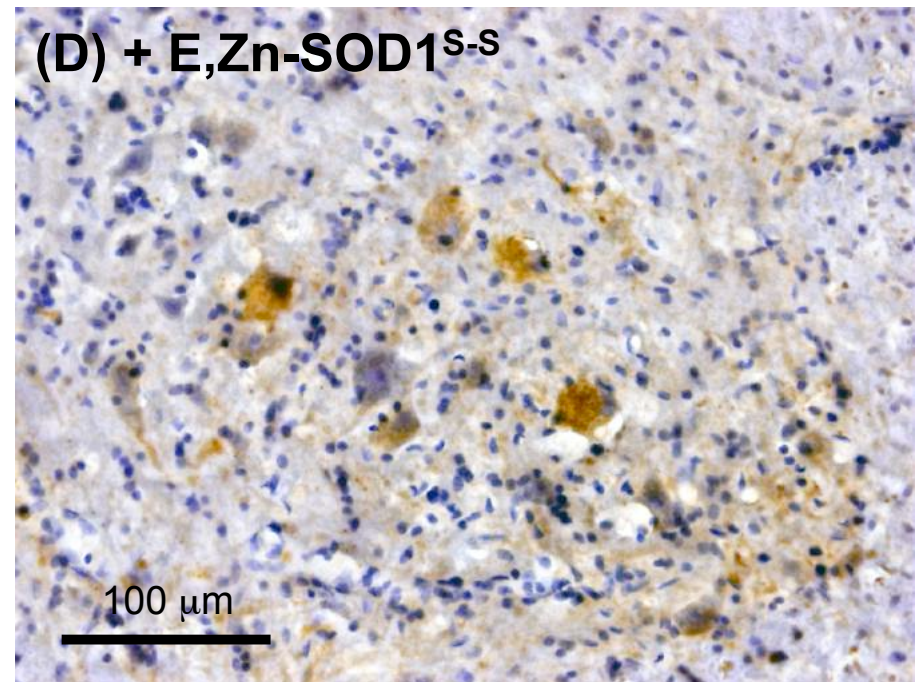

Supplement: Additional file 3: Figure S2. — Specificity of anti-SOD1olig antibody for immunohistochemical examination of mouse spinal cords. The sections of lumbar spinal cords of (A, B) a non-transgenic mouse (C57BL/6) at 200 days of age and (C, D) a G1H mouse at 100 days of age were stained with anti-SOD1olig antibody. For the absorption experiments shown in (C, D), the anti-SOD1olig antibody was pre-absorbed on ice for 5 h with either (C) soluble disulfide-crosslinked SOD1(A4V) oligomers (6.7 μM in monomer base) or (D) E,Zn-SOD1S-S (6.7 μM). Nuclei were also counterstained with hematoxylin (blue). (PDF 265 kb) [file 13024_2016_145_MOESM3_ESM.pdf]

**(A) 60 days**

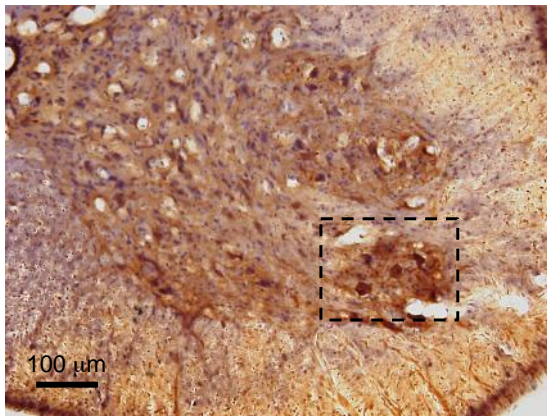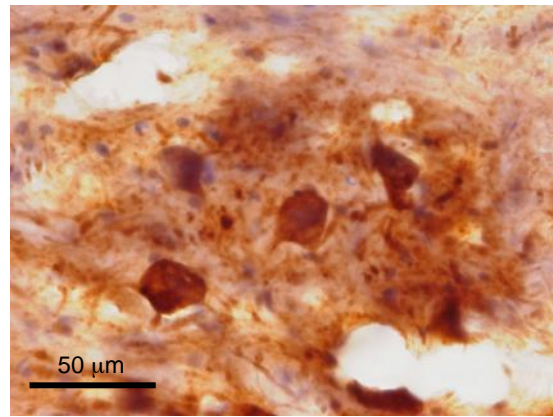

**(B) 100 days**

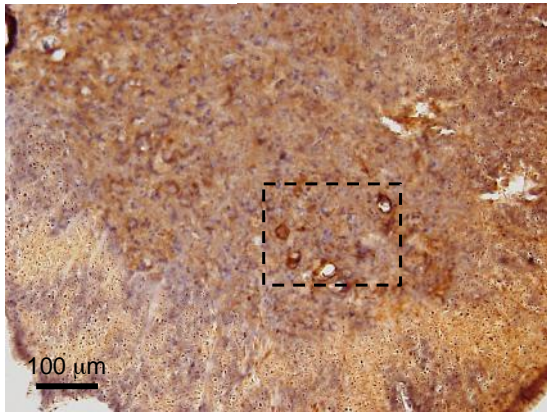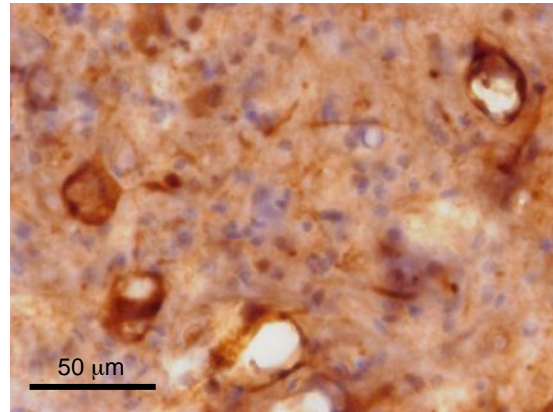

**(C) 140 days**

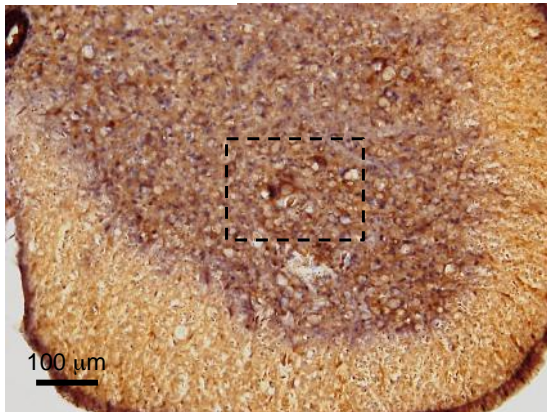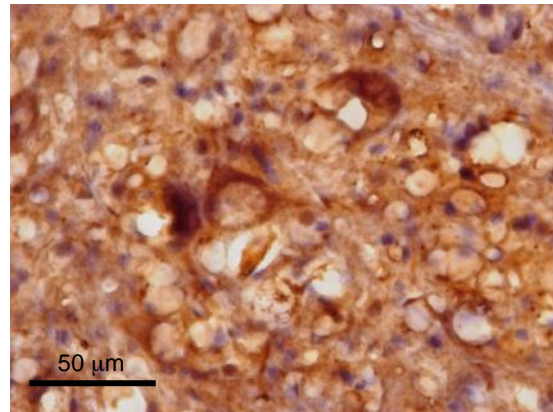

**(D) 160 days**

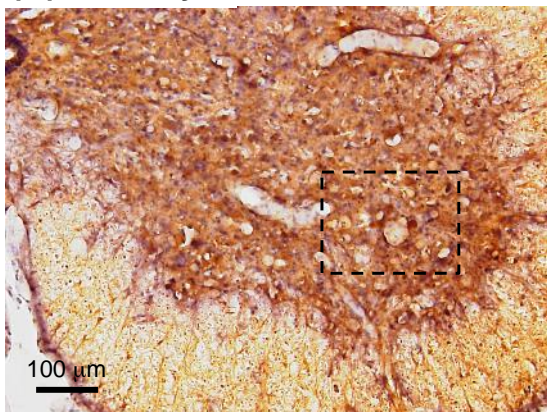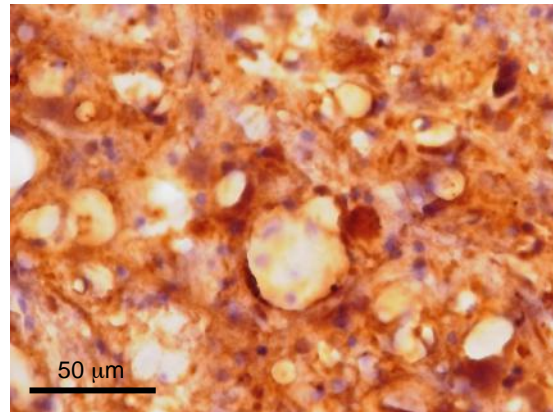

Supplement: Additional file 4: Figure S3. — Immunohistochemical examination on lumbar spinal cords of G1H mice with anti-SOD1 antibody. The sections of lumbar spinal cords of G1H mice at (A) 60, (B) 100, (C) 140, and (D) 160 days of age were stained with monoclonal anti-SOD1 (clone 1G2, MBL) antibody. The images in the low magnification are shown in the left panel, where the region enclosed with a broken line is magnified and shown in the right panel. Nuclei were counterstained with hematoxylin (blue). The bar in each panel represents 100 μm (left panel) and 50 μm (right panel). (PDF 273 kb) [file 13024_2016_145_MOESM4_ESM.pdf]

(A)

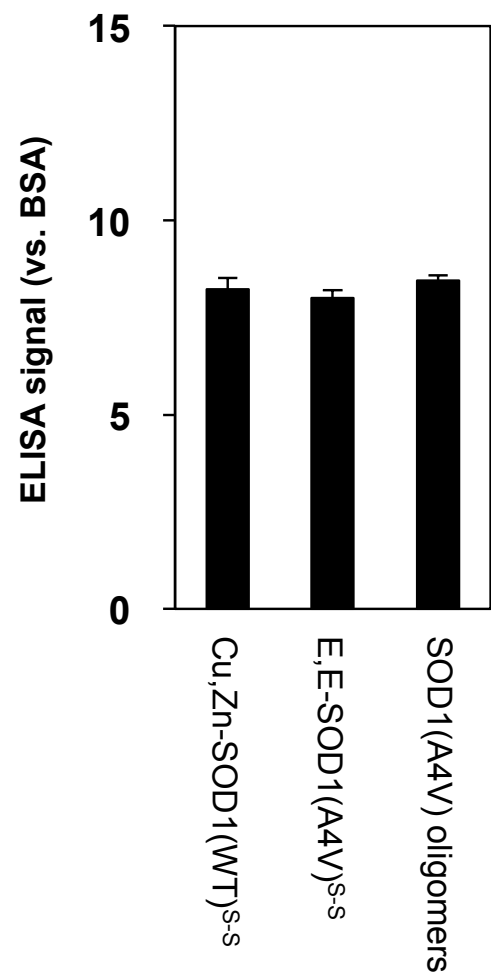

(B)

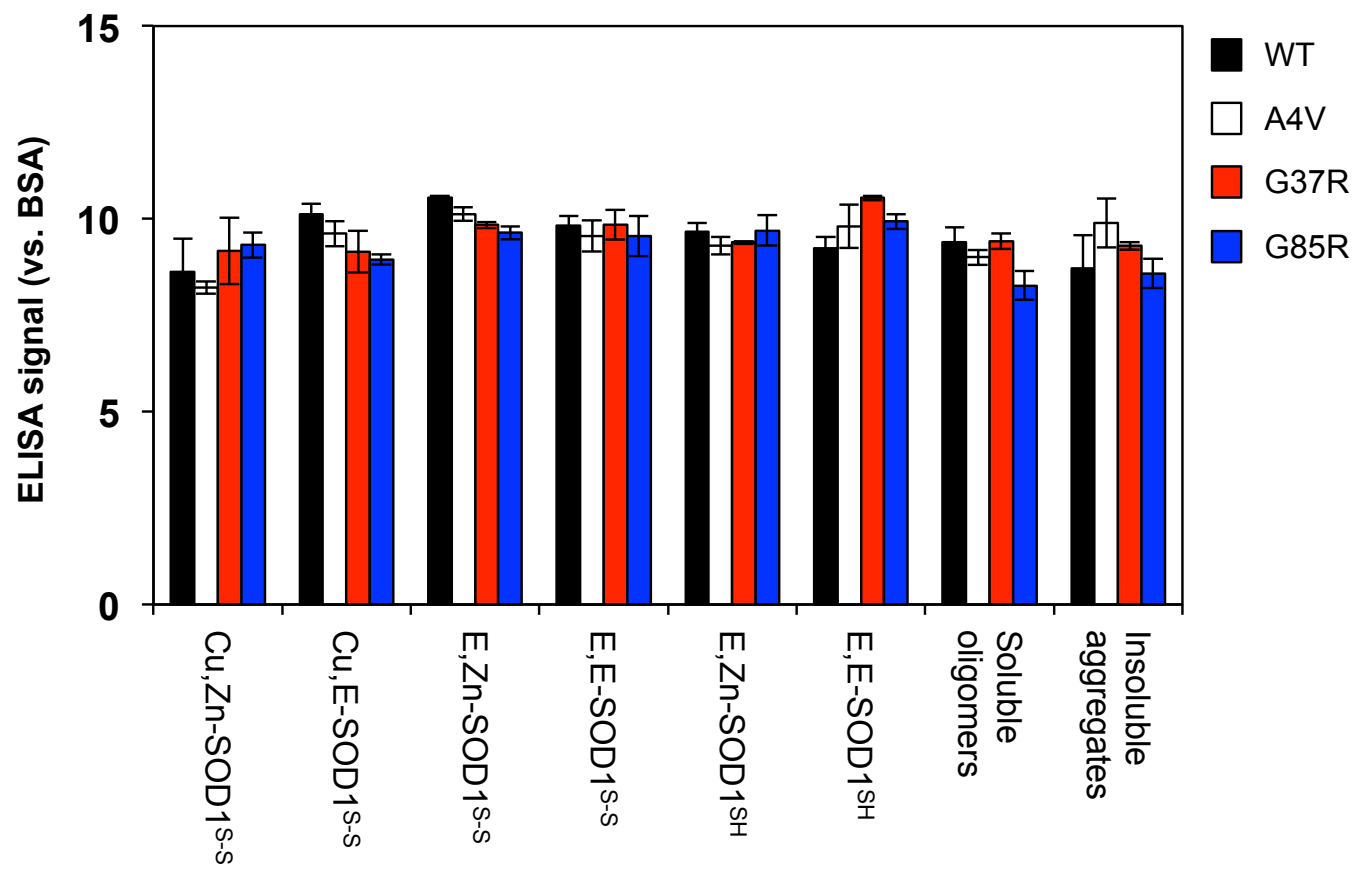

Supplement: Additional file 6: Figure S5. — Equal fixation of various forms of SOD1 proteins on the ELISA plate. Amounts of SOD1 proteins fixed on the ELISA plates for the experiments in Fig. 5 were quantified by indirect ELISA with polyclonal anti-SOD1 antibody (FL-154, Santa Cruz Biotechnology). Amounts of SOD1 in the plates for experiments in Fig. 5a were shown in the panel (A), while those in Fig. 5b, c, and d were in the panel (B). The ELISA signal was represented as a ratio against that obtained using BSA. Three independent experiments were performed to estimate error bars (standard deviation). No statistically significant difference in the ELISA signal was confirmed among the in vitro samples examined. (PDF 52 kb) [file 13024_2016_145_MOESM6_ESM.pdf]

**(A) USOD-like**

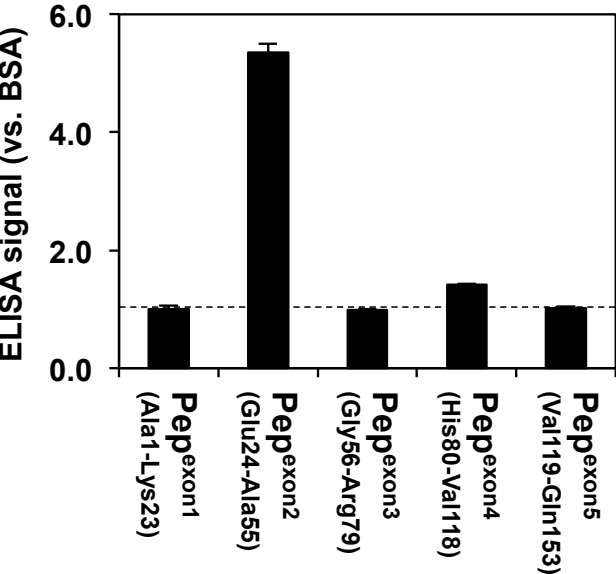

**(B) SEDI-like**

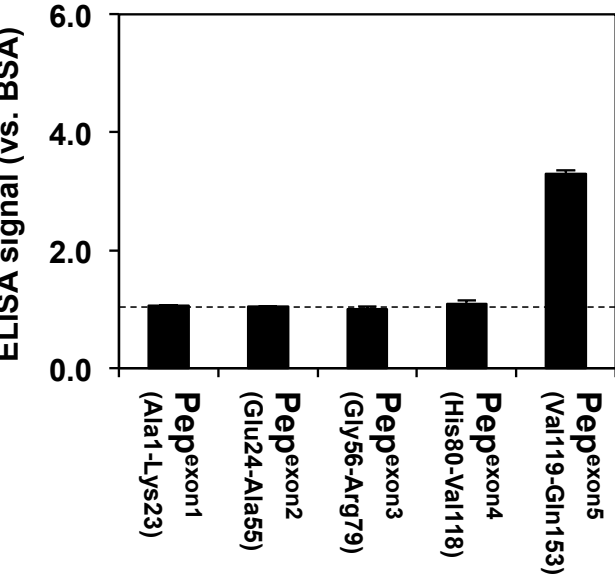

**(C) anti-GST**

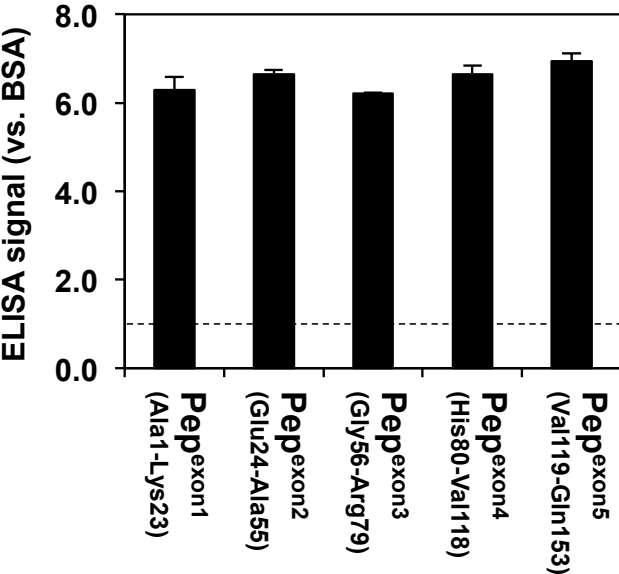

Supplement: Additional file 7: Figure S6. — Epitope analysis on USOD-like and SEDI-like antibodies by indirect ELISA. The peptides were prepared as a fusion protein with an N-terminal 6x His tagged GST and adsorbed on an ELISA plate. SOD1 species recognized by (A) USOD-like and (B) SEDI-like antibodies were quantified as ELISA signals that were represented as a ratio against those of BSA. Almost equal amounts of peptides were adsorbed on the plate, which was confirmed by an ELISA using anti-GST antibody (C). Three independent experiments were performed to estimate error bars (standard deviation). (PDF 29 kb) [file 13024_2016_145_MOESM7_ESM.pdf]

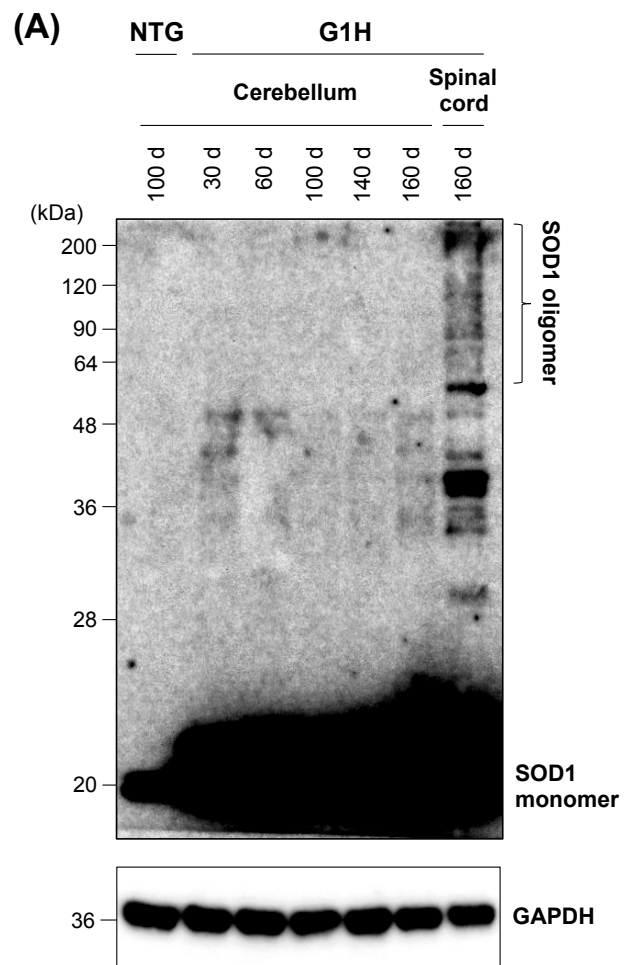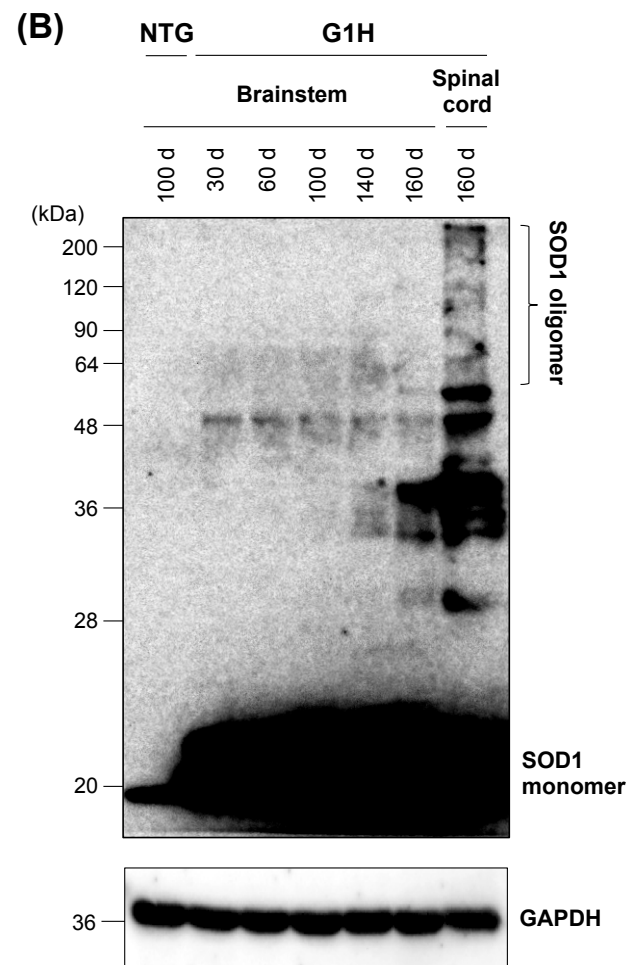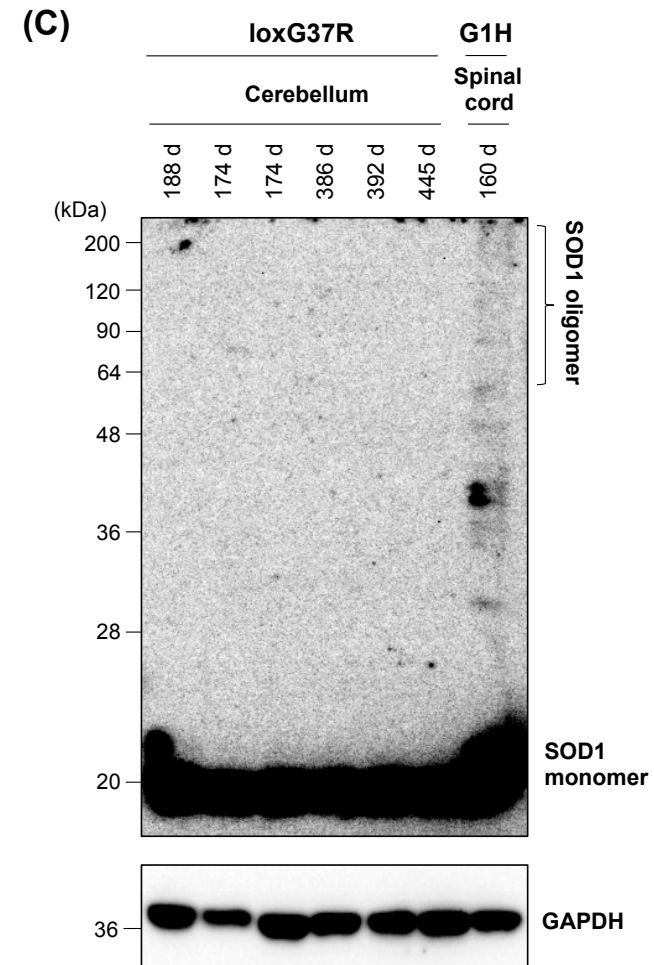

Supplement: Additional file 9: Figure S8. — Examination of soluble SOD1 disulfide-crosslinked oligomers in cerebellum and brainstem of ALS-model mice. (A) Cerebellum and (B) brainstem of non-transgenic (NTG) and G1H mice and (C) cerebellum of loxG37R mice were homogenized in the presence of 100 mM iodoacetamide and 1% NP-40 and centrifuged at 20,000 x g for 30 min so as to prepare soluble supernatant. The supernatant was then separated in a polyacrylamide gel by non-reducing SDS-PAGE and probed by Western blot using anti-SOD1 antibody (FL-154, Santa Cruz Biotechnology). For comparison, the soluble fraction of the lumbar spinal cord homogenates of G1H mice at 160 days of age was also loaded on the same gel. GAPDH was used as a protein loading control for Western blot. (PDF 812 kb) [file 13024_2016_145_MOESM9_ESM.pdf]

**(A) anti-SOD1<sup>int</sup>**

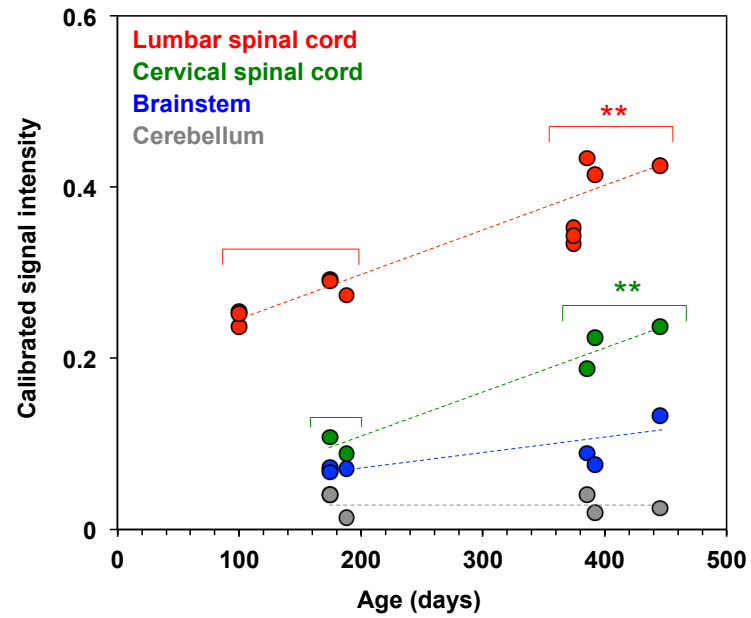

**(B) anti-SOD1 (FL-154)**

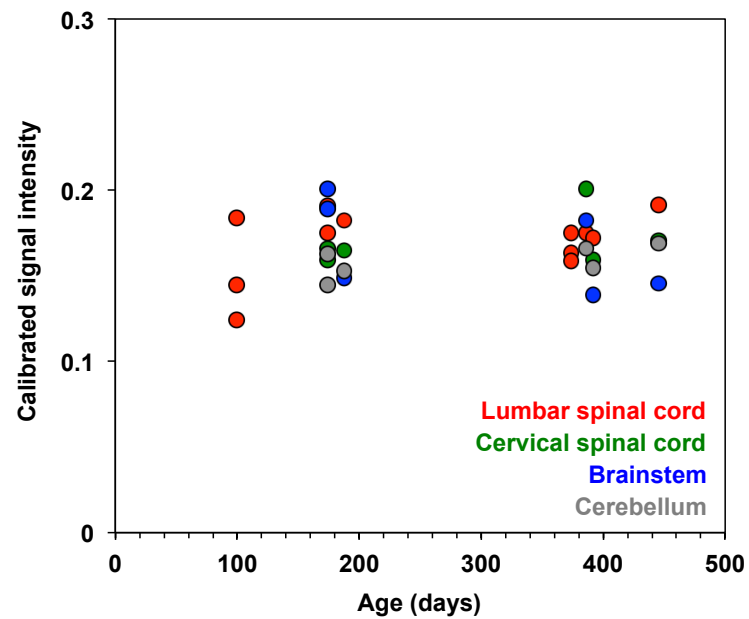

**(C)**

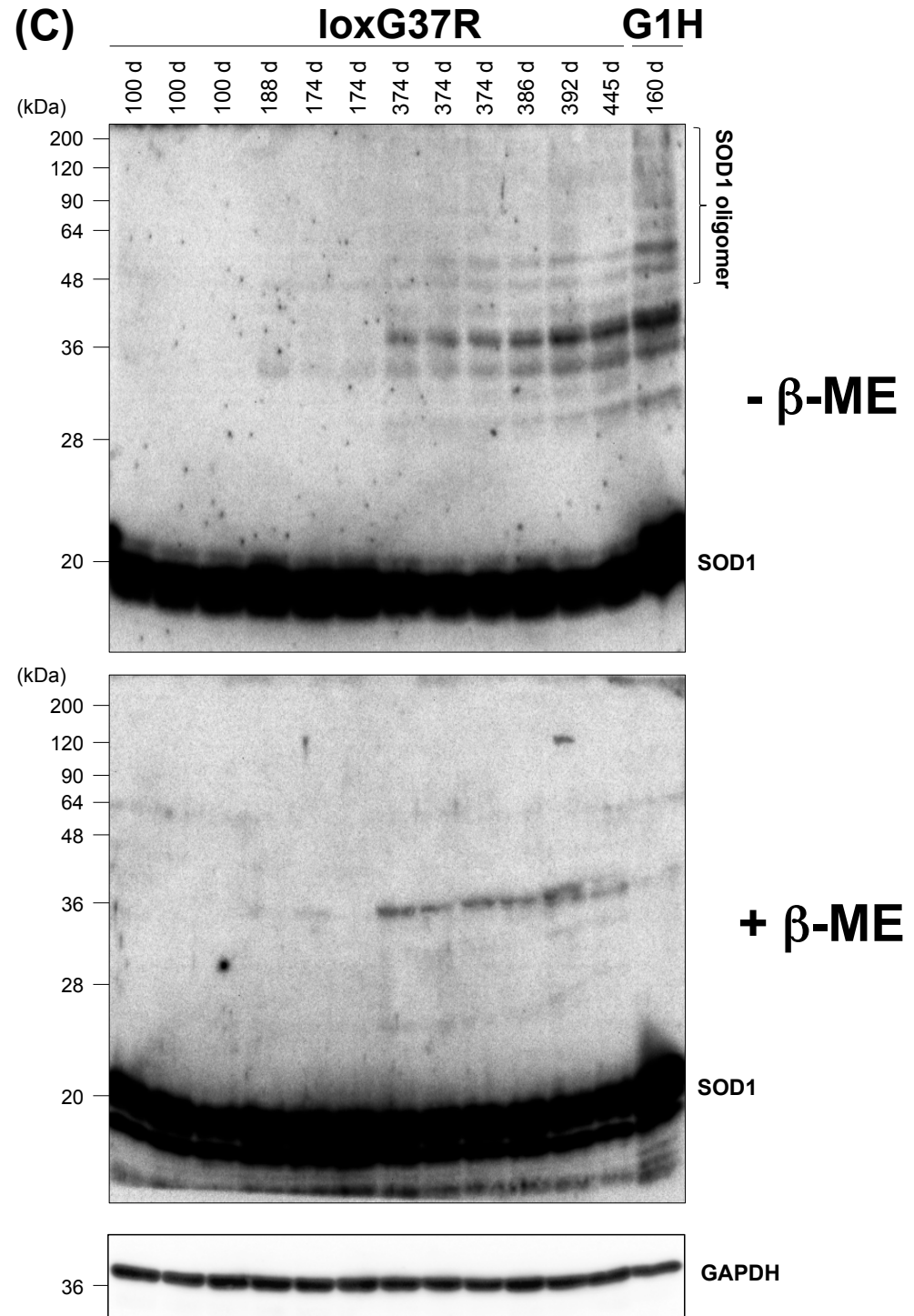

Supplement: Additional file 10: Figure S9. — Anti-SOD1int antibody specifically detects pathological SOD1 in spinal cords of loxG37R mice. (A, B) SOD1 species recognized by (A) anti-SOD1int and (B) anti-SOD1 (FL-154, Santa Cruz Biotechnology) antibody were quantified in the soluble fraction of the homogenates of lumbar spinal cord (red), cervical spinal cord (green), brainstem (blue), and cerebellum (gray) of loxG37R mice by sandwich ELISA. Ages of the mouse samples examined are as follows; 100 (three independent mice), 174 (two independent mice), 188, 374 (three independent mice), 386, 392, and 445 days of age. The data were divided into two groups before and after the onset of the disease (350 days of age) and statistically analyzed by two-tailed student’s t test. The difference in the signal intensity before and after the disease onset was statistically significant in lumbar and cervical spinal cords (**: P < 0.01). (C) Soluble disulfide-crosslinked SOD1 oligomers in loxG37R mice were examined by Western blotting. Lumbar spinal cords of loxG37R and G1H mice at indicated days of ages were homogenized in the presence of 100 mM iodoacetamide and 1% NP-40 and centrifuged at 20,000 x g for 30 min so as to prepare soluble supernatant. In the presence and absence of the reducing reagent, β-ME, the supernatant was then separated in a polyacrylamide gel by SDS-PAGE and probed by Western blot using anti-SOD1 antibody (FL-154, Santa Cruz Biotechnology). GAPDH was used as a protein loading control for Western blot. (PDF 870 kb) [file 13024_2016_145_MOESM10_ESM.pdf]

**(A) anti-SOD1<sup>int</sup>**

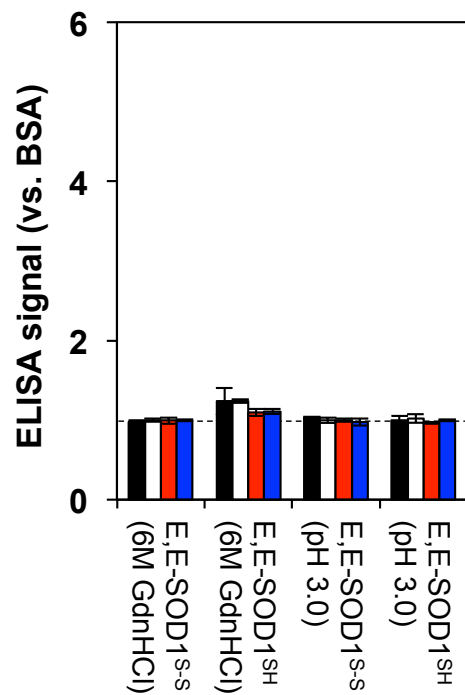

**(B) USOD-like**

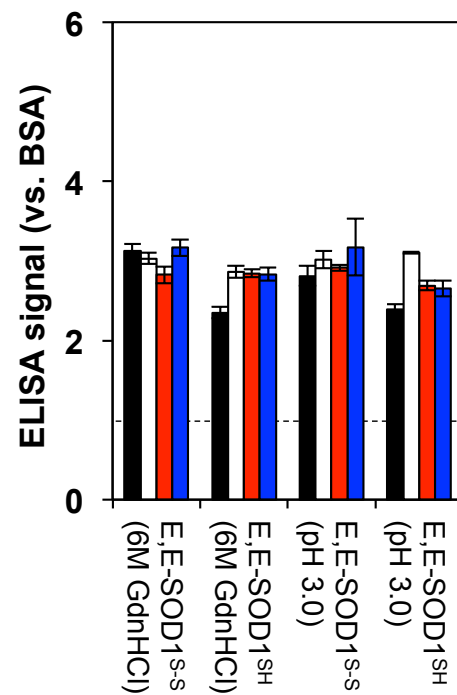

**(C) SEDI-like**

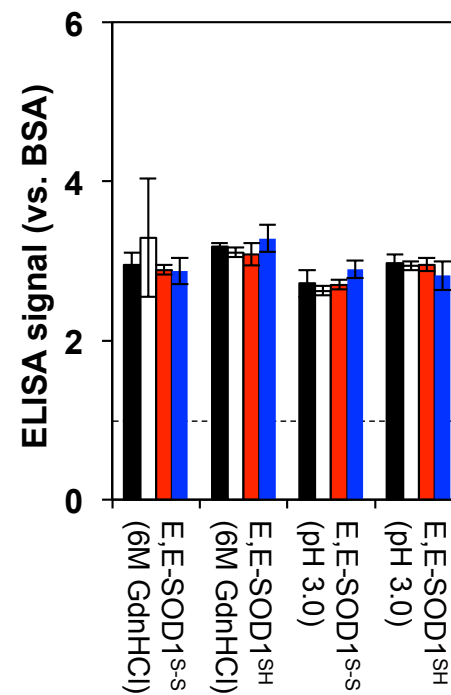

**(D) anti-SOD1 (FL-154)**

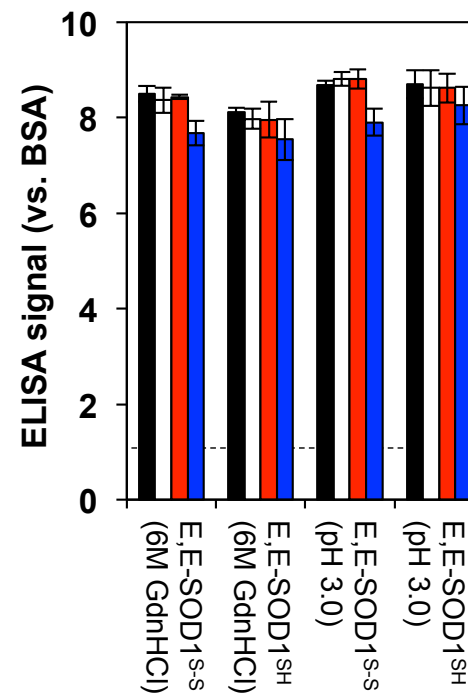

■ WT    □ A4V    ■ G37R    ■ G85R

Supplement: Additional file 11: Figure S10. — Reactivity of the antibodies toward chemically misfolded/unfolded forms of SOD1. E,E-SOD1SH and E,E-SOD1S-S (100 μM; WT, A4V, G37R, and G85R) were first incubated at room temperature for two hours either in 50 mM Tris/100 mM NaCl/5 mM EDTA/6 M guanidine hydrochloride (GdnHCl) at pH 7.4 or in 50 mM sodium acetate buffer at pH 3.0 and then fixed on an ELISA plate. ELISA was performed using (A) anti-SOD1int, (B) USOD-like, (C) SEDI-like, and (D) anti-SOD1 (FL-154, Santa Cruz Biotechnology) antibodies. The ELISA signal was represented as a ratio against that obtained using BSA. Three independent experiments were performed to estimate error bars (standard deviation). (PDF 49 kb) [file 13024_2016_145_MOESM11_ESM.pdf]
