# Supplementary material for: Immunochemical characterization on pathological oligomers of mutant Cu/Zn-superoxide dismutase in amyotrophic lateral sclerosis
Source: Mol Neurodegener. 2017 Jan 5;12:2. doi: 10.1186/s13024-016-0145-9 (PMC5216565; doi:10.1186/s13024-016-0145-9)
Supplement: Additional file 5: Figure S4. — Representative images for immunohistochemical examination of non SOD1-ALS cases. Spinal cord sections of (A) non-ALS (C1 in Additional file 1: Table S1), (B) non-ALS (C3 in Additional file 1: Table S1), (C) sporadic ALS (sALS3 in Additional file 1: Table S1), and (D) sporadic ALS (sALS4 in Additional file 1: Table S1) cases were immunostained with either (A) anti-SOD1olig or (B-D) anti-SOD1int antibody. Nuclei were also stained by hematoxylin (blue). The bars represent 50 μm. (PDF 1002 kb) [file 13024_2016_145_MOESM5_ESM.pdf]

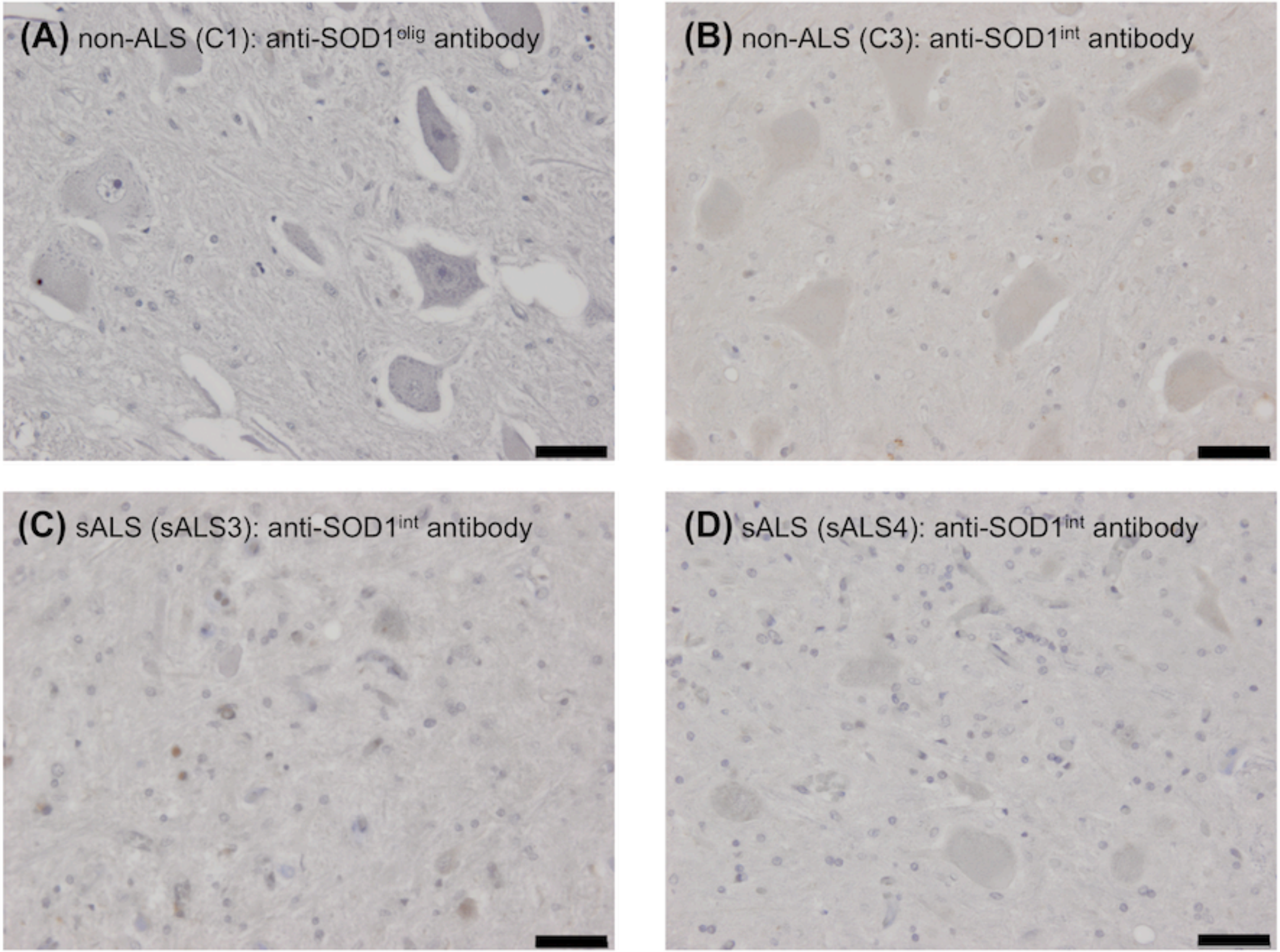
(A) non-ALS (C1): anti-SOD1^{olig} antibody
(B) non-ALS (C3): anti-SOD1^{int} antibody
(C) sALS (sALS3): anti-SOD1^{int} antibody
(D) sALS (sALS4): anti-SOD1^{int} antibody
