# Supplementary material for: Immunochemical characterization on pathological oligomers of mutant Cu/Zn-superoxide dismutase in amyotrophic lateral sclerosis
Source: Mol Neurodegener. 2017 Jan 5;12:2. doi: 10.1186/s13024-016-0145-9 (PMC5216565; doi:10.1186/s13024-016-0145-9)
Supplement: Additional file 8: Figure S7. — Anti-SOD1int antibody specifically detects pathological SOD1 in spinal cords of ALS-model mice. (A, B) SOD1 species recognized by (A) anti-SOD1int and (B) anti-SOD1 (FL-154, Santa Cruz Biotechnology) antibody were quantified in the soluble fraction of the homogenates of lumbar spinal cords of non-transgenic (nonTG), WT, and G1H mice by sandwich ELISA. The data on G1H mice at 30 days of age are the same with those in Fig. 6a and b and shown here again for comparison. (C, D) Sandwich ELISA with (C) anti-SOD1int and (D) anti-SOD1 (FL-154, Santa Cruz Biotechnology) antibody was examined by using the soluble fraction of the homogenates of lumbar spinal cords of G1H mice. The samples were pre-treated with 10 mM DTT for reducing disulfide bonds. In all panels, three independent mouse samples were examined to estimate error bars (standard deviation), and the statistical analysis has been performed to obtain the P values indicated in the figures. (PDF 47 kb) [file 13024_2016_145_MOESM8_ESM.pdf]

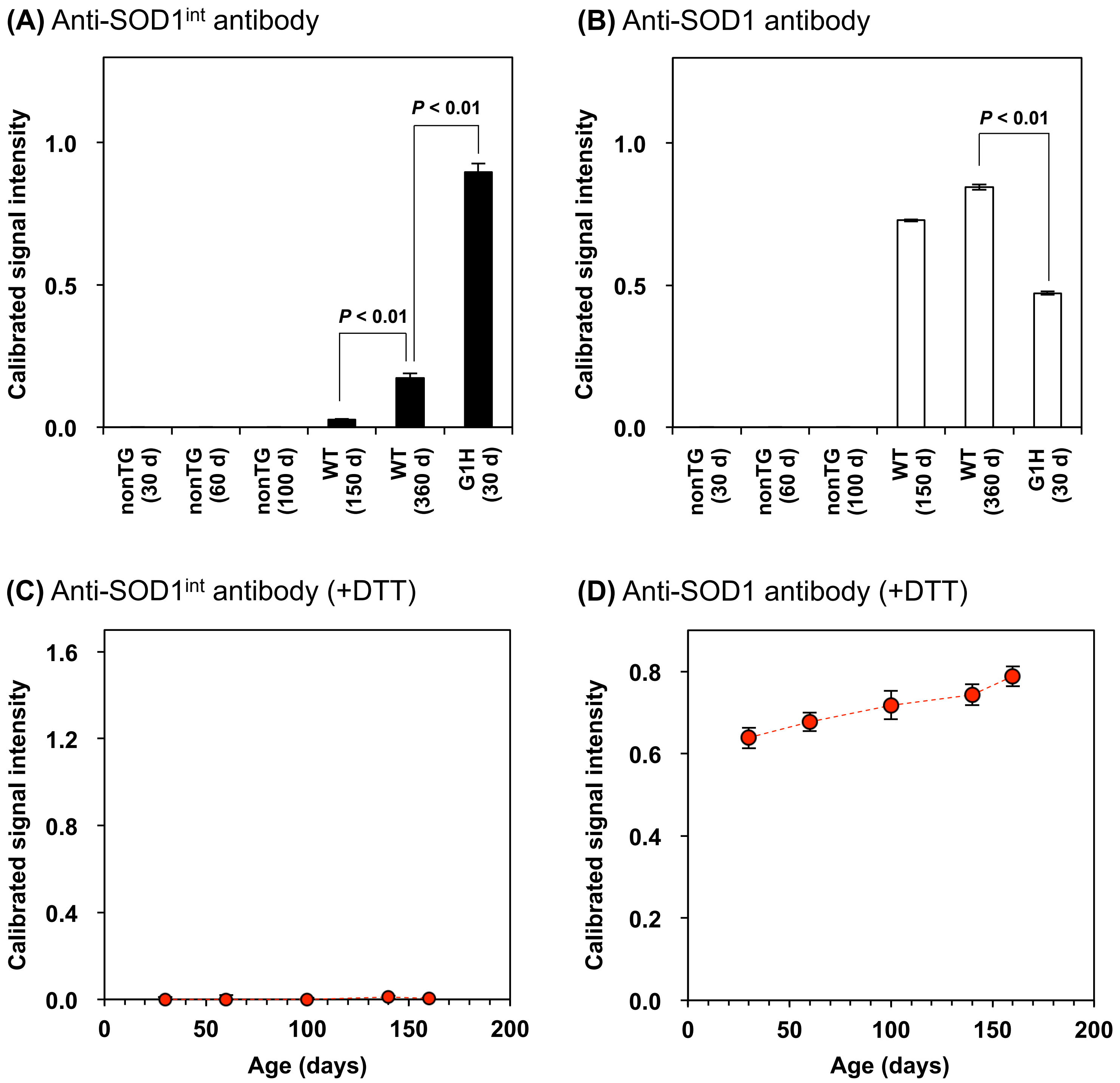

(A) Anti-SOD1int antibody
(B) Anti-SOD1 antibody
(C) Anti-SOD1int antibody (+DTT)
(D) Anti-SOD1 antibody (+DTT)
Calibrated signal intensity
P < 0.01
nonTG (30 d)
nonTG (60 d)
nonTG (100 d)
WT (150 d)
WT (360 d)
G1H (30 d)
Age (days)
0.0
0.5
1.0
0.4
0.8
1.2
1.6
0.2
0.6
0
50
100
150
200
